# Progress in reducing inequalities in cardiovascular disease mortality in Europe

**DOI:** 10.1136/heartjnl-2019-315129

**Published:** 2019-08-22

**Authors:** Chiara Di Girolamo, Wilma J Nusselder, Matthias Bopp, Henrik Brønnum-Hansen, Giuseppe Costa, Katalin Kovács, Mall Leinsalu, Pekka Martikainen, Barbara Pacelli, José Rubio Valverde, Johan P Mackenbach

**Affiliations:** 1 Department of Medical and Surgical Sciences, University of Bologna, Bologna, Italy; 2 Department of Public Health, Erasmus Medical Center, Rotterdam, Netherlands; 3 Epidemiology, Biostatistics and Prevention Institute, University of Zurich, Zurich, Switzerland; 4 Department of Public Health, University of Copenhagen, Copenhagen, Denmark; 5 Department of Clinical Medicine and Biology, University of Turin, Torino, Italy; 6 Demographic Research Institute, Budapest, Hungary; 7 Stockholm Centre for Health and Social Change, Södertörn University, Huddinge, Sweden; 8 Department of Epidemiology and Biostatistics, National Institute for Health Development, Tallin, Estonia; 9 Population Research Unit, University of Helsinki, Helsinki, Finland; 10 Regional Health and Social Care Agency of Emilia-Romagna, Bologna, Italy

**Keywords:** cardiovascular diseases, mortality, inequalities, socioeconomic position, europe

## Abstract

**Objective:**

To assess whether recent declines in cardiovascular mortality have benefited all socioeconomic groups equally and whether these declines have narrowed or widened inequalities in cardiovascular mortality in Europe.

**Methods:**

In this prospective registry-based study, we determined changes in cardiovascular mortality between the 1990s and the early 2010s in 12 European populations by gender, educational level and occupational class. In order to quantify changes in the magnitude of differences in mortality, we calculated both ratio measures of relative inequalities and difference measures of absolute inequalities.

**Results:**

Cardiovascular mortality has declined rapidly among lower and higher socioeconomic groups. Relative declines (%) were faster among higher socioeconomic groups; absolute declines (deaths per 100 000 person-years) were almost uniformly larger among lower socioeconomic groups. Therefore, although relative inequalities increased over time, absolute inequalities often declined substantially on all measures used. Similar trends were seen for ischaemic heart disease and cerebrovascular disease mortality separately. Best performer was England and Wales, which combined large declines in cardiovascular mortality with large reductions in absolute inequalities and stability in relative inequalities in both genders. In the early 2010s, inequalities in cardiovascular mortality were smallest in Southern Europe, of intermediate magnitude in Northern and Western Europe and largest in Central-Eastern European and Baltic countries.

**Conclusions:**

Lower socioeconomic groups have experienced remarkable declines in cardiovascular mortality rates over the last 25 years, and trends in inequalities can be qualified as favourable overall. Nevertheless, further reducing inequalities remains an important challenge for European health systems and policies.

## Introduction

Cardiovascular diseases are one of the leading causes of death in Europe where they are responsible for approximately 4 million deaths yearly (about 45% of the total deaths).^1^ Ischaemic heart disease (IHD) and cerebrovascular disease (CVD), the two main groups of cardiovascular diseases, account for about 2 million and 1 million deaths, respectively.^2^ Cardiovascular mortality decreased remarkably across Europe over the last three decades. However, these favourable trends were not observed evenly across all geographic areas, and an East–West divide has been reported.^3^

Studies that explored socioeconomic differentials in cardiovascular mortality in Europe in the 1980s and the 1990s reported a North–South gradient with larger relative inequalities in Northern than in Southern European countries.^4 5^ This North–South divide was mainly driven by differences in mortality from IHD, whereas CVD inequalities did not show clear geographical patterns. Analyses of temporal changes in cardiovascular mortality over the 1980s and 1990s highlighted that, while relative inequalities in IHD generally widened over time across countries in Western Europe, they remained fairly stable in CVD mortality.^6^ Previous studies have paid less attention to what happened to absolute inequalities.

As cardiovascular mortality has continued to decline rapidly, and a recent update of socioeconomic inequalities in cardiovascular mortality in Europe is lacking, the aim of this study is to assess whether these declines in mortality have benefited all socioeconomic groups equally and whether they have narrowed or widened absolute and relative inequalities in cardiovascular, IHD and CVD mortality in 12 European populations.

## Methods

### Data

Mortality data were obtained from official registers for 12 European populations, encompassing Nordic (Finland and Denmark) and Western countries (England and Wales, Austria and Switzerland), Southern populations (Spain: Barcelona, Italy: Turin and Emilia), Central-Eastern (Hungary and Poland) and Baltic countries (Estonia and Lithuania) and covering a period between approximately 1990 and 2014. For most countries, data came from a longitudinal mortality follow-up after a census in which socioeconomic information were obtained from the census, and the subjects were followed up to death, emigration or new census. All data covered complete national populations with the exceptions of Italy (data only available for Turin and three towns in the Emilia region: Bologna, Modena and Reggio Emilia), Spain (data only available for Barcelona) and England and Wales (1% sample of the national population). Data sources’ characteristics are presented in online supplementary table S1. Data were centrally harmonised to enhance comparability and consisted of cause-specific deaths and person-years counts by gender, 5-year age group and socioeconomic position (SEP) (online supplementary table S3–S5).

10.1136/heartjnl-2019-315129.supp1 Supplementary data

SEP was indicated by educational level and occupational class. The highest education attained was classified according to the International Standard Classification of Education (ISCED) and grouped into three categories: low (ISCED 0–2), middle (ISCED 3–4) and high education (ISCED 5–6). For England and Wales, detailed information was not available for the 1991 census, and therefore, low and middle education were combined. Occupational class was classified following the Erikson-Goldthorpe-Portocarero scheme^7^ and was grouped into five classes: upper non-manual employees, lower non-manual employees, manual workers, farmers and self-employed. Information on occupational class was available in 8 of the 12 populations (online supplementary table S1); because it is less reliable for women, only occupational inequalities among men are reported.

The underlying causes of deaths were coded according to the International Classification of Diseases and grouped into total cardiovascular disease, IHD and CVD (codes detailed in online supplementary table S2).

### Statistical analysis

Analyses by education were restricted to subjects aged 35–79 years and those by occupation to men aged 35–64 years.

Mortality rates were directly age-standardised with reference to the 2013 European standard population^8^ and calculated separately by sex, SEP, population and period. To correct for the between-country differences in the length of the follow-up, average absolute (deaths per 100 000 person-years) and relative (%) changes per year in the age-standardised mortality rates (ASMRs) by education and occupation between beginning and end of the observation periods were estimated.

Differences in mortality by SEP were assessed using relative and absolute measures of inequalities. We used three sets of quantitative measures, in order to capture various aspects. (1) In the analyses by education, relative inequalities were estimated with the Relative Index of Inequality (RII) and absolute inequalities with the Slope Index of Inequality (SII). These summary regression-based indexes, which allow comparisons over time and across populations with different educational distributions and require an unambiguous ordering of groups, were estimated through Poisson models. The RII and the SII quantify the socioeconomic gradient and correspond to the expected relative and excess risks comparing the hypothetical very lowest and very highest educational positions.^9^ (2) In the analyses by occupation, whose classification is not strictly hierarchical, relative and absolute inequalities were quantified using the Average Intergroup Difference (AID).^10^ The absolute version of the AID is defined as the population-weighted average of mortality differences between occupational-class-specific mortality rates across all possible group-specific pairs (number of occupational classes are detailed in online supplementary table S1). The relative version of the AID is obtained by dividing the absolute AID by the population-weighted average of the group-specific mortality and then multiplying it by 100. (3) For both SEP indicators, the population impact of differences in mortality was estimated from the ASMRs using the population attributable risk (PAR) and the population attributable fraction (PAF), which are measures widely used in public health assessments to estimate the expected population impact of changing the distribution of risk factors in that population. The PAR and PAF quantify the absolute and relative number of deaths in the whole population that could be prevented had everyone in the population the same level of mortality as those in the highest SEP. The 95% CIs for the average changes, the PAF and the PAR were obtained using bootstrapping of 1000 replicas.

In the calculation of mortality differentials by occupational class, a correction algorithm was applied to the populations for whom information on occupational class among inactive men was lacking, in order to minimise the potential for the underestimation of mortality differences. This methodology, which has been described elsewhere,^11^ is based on the proportional distribution in the population and the relative mortality level of the inactive. Results by occupation are commented in the text but only reported in the supplementary material.

## Results

In this study, 2 152 018 deaths occurred over 504 631 113 person-years of follow-up.

### Trends in mortality by education

**Figure 1:**
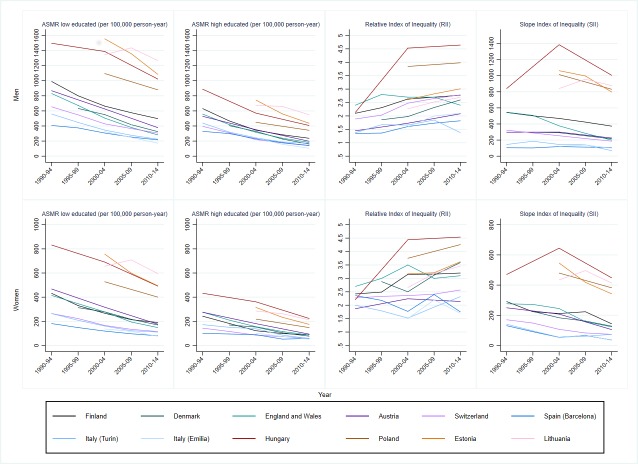
Trends in total cardiovascular disease mortality (ASMR, age-standardised mortality rate) and inequalities (Relative and Slope Index of Inequality) by educational level, population and gender, 35–79 years.

Since the 1990s, there was a dramatic decline in total cardiovascular, IHD and CVD mortality among low and high educated men and women in Nordic, Western and Southern European populations (figure 1, online supplementary figure S1–S2). Central-Eastern European and Baltic countries also experienced a mortality decrease with the exception of Lithuania, where ASMRs increased until the end of the 2000s, especially among the low educated, and declined afterwards.

Estimates of the average changes in total cardiovascular, IHD and CVD mortality by education showed that generally, in both genders, absolute declines were larger among the low educated, while relative declines were larger among the high educated (table 1). However, there were a few exceptions. Larger absolute and relative declines among the high educated (or even an increase in mortality among the low educated) consistently occurred in Lithuania. Conversely, relative declines were not clearly larger among the high educated for CVD mortality among men in Turin (Italy) and all groups of causes among women in Barcelona (Spain).

**Table 1:** Average relative and absolute changes per year in age−standardised mortality rates (95% CI) between 2010-2014 and 1990-1994 (or as otherwise specified) for cardiovascular, ischaemic heart and cerebrovascular disease mortality, by low and high education, population and gender, 35–79 years

	Cardiovascular disease		Ischaemic heart disease		Cerebrovascular disease	
	Relative*	Absolute†	Relative*	Absolute†	Relative*	Absolute†
Men						
Finland						
Low	−3.6 (−3.6 to −3.5)	−26.0 (−26.1 to −25.8)	−4.2 (−4.3 to −4.2)	−19.7 (−19.9 to −19.6)	−3.9 (−4.0 to −3.8)	−5.0 (−5.0 to −4.9)
High	−5.0 (−5.0 to −5.0)	−20.7 (−21.4 to −19.9)	−5.7 (−5.7 to −5.7)	−14.5 (−15.0 to −13.9)	−5.3 (−5.4 to −5.3)	−4.4 (−4.7 to −4.1)
** **Denmark‡						
Low	−4.3 (−4.4 to −4.3)	−20.4 (−26.8 to −24.1)	−6.4 (−6.5 to −6.4)	−15.1 (−15.4 to −14.9)	−2.9 (−3.0 to −2.8)	−2.7 (−2.8 to −2.6)
High	−6.0 (−6.0 to −5.9)	−16.3 (−17.0 to −15.5)	−8.3 (−8.4 to −8.2)	−10.2 (−10.9 to −9.7)	−5.0 (−5.1 to −4.9)	−2.9 (−3.3 to −2.6)
England and Wales						
Low	−5.8 (−6.1 to −5.6)	−30.4 (−38.8 to −37.2)	−6.1 (−6.4 to −5.8)	−21.3 (−21.6 to −21.1)	−6.5 (−7.1 to −5.8)	−5.2 (−5.4 to −5.0)
High	−6.0 (−6.3 to −5.7)	−20.9 (−22.5 to −18.9)	−6.0 (−6.3 to −5.7)	−13.5 (−14.8 to −12.1)	−6.5 (−7.6 to −4.9)	−3.6 (−4.5 to −2.7)
Austria						
Low	−4.1 (−4.1 to −4.0)	−24.5 (−25.1 to −23.8)	−3.6 (−3.7 to −3.6)	−11.0 (−11.5 to −10.7)	−5.7 (−5.8 to −5.6)	−6.6 (−7.0 to −6.3)
High	−4.8 (−5.0 to −4.6)	−16.5 (−18.4 to −14.5)	−4.7 (−4.9 to −4.4)	−8.9 (−10.3 to −7.6)	−6.2 (−6.6 to −5.9)	−3.9 (−4.8 to −3.1)
Switzerland						
Low	−3.9 (−4.0 to −3.8)	−18.3 (−18.4 to −18.2)	−4.2 (−4.4 to −4.1)	−9.6 (−9.6 to −9.5)	−4.9 (−5.1 to −4.6)	−3.1 (−3.2 to −3.0)
High	−5.3 (−5.4 to −5.3)	−13.4 (−13.8 to −13.1)	−6.1 (−6.1 to −6.0)	−7.8 (−8.1 to −7.5)	−5.6 (−5.8 to −5.5)	−2.0 (−2.2 to −1.9)
Spain (Barcelona)						
Low	−3.5 (−3.6 to −3.3)	−10.6 (−10.7 to −10.5)	−3.7 (−4.0 to −3.5)	−4.8 (−4.9 to −4.7)	−5.3 (−5.6 to −4.9)	−3.6 (−3.7 to −3.6)
High	−4.6 (−4.7 to −4.4)	−10.3 (−10.9 to −9.9)	−4.9 (−5.2 to −4.5)	−5.0 (−5.3 to −4.6)	−5.4 (−5.8 to −4.9)	−2.7 (−3.0 to −2.5)
Italy (Turin)						
Low	−4.9 (−5.2 to −4.7)	−18.6 (−18.9 to −18.3)	−4.5 (−4.8 to −4.1)	−7.0 (−7.2 to −6.8)	−6.0 (−6.4 to −5.4)	−5.4 (−5.5 to −5.2)
High	−5.1 (−5.5 to −4.6)	−14.9 (−15.7 to −13.9)	−4.9 (−5.5 to −4.2)	−6.0 (−6.6 to −5.4)	−4.6 (−5.3 to −3.3)	−2.8 (−3.2 to −2.5)
Italy (Emilia)§						
Low	−6.1 (−6.4 to −5.9)	−15.3 (−15.6 to −15.0)	−7.5 (−7.9 to −7.1)	−9.0 (−9.4 to −8.7)	−5.9 (−6.5 to −5.3)	−2.7 (−2.8 to −2.5)
High	−7.9 (−8.3 to −7.6)	−13.6 (−15.0 to −12.2)	−9.3 (−10.0 to −8.6)	−7.1 (−8.1 to −6.1)	−8.0 (−8.6 to −7.0)	−2.9 (−3.5 to −2.3)
Hungary						
Low	−1.8 (−1.8 to −1.8)	−22.6 (−22.7 to −22.6)	−1.0 (−1.0 to −1.0)	−5.8 (−5.8 to −5.7)	−3.0 (−3.0 to −2.9)	−9.3 (−9.4 to −9.3)
High	−3.6 (−3.7 to −3.6)	−22.9 (−23.4 to −22.3)	−3.5 (−3.5 to −3.5)	−12.4 (−12.8 to −12.0)	−4.5 (−4.6 to −4.4)	−5.5 (−5.8 to −5.2)
Poland§						
Low	−2.4 (−2.4 to −2.4)	−24.0 (−24.1 to −23.9)	−4.1 (−4.1 to −4.0)	−14.2 (−14.2 to −14.1)	−3.7 (−3.7 to −3.7)	−7.8 (−7.9 to −7.8)
High	−2.9 (−3.0 to −2.9)	−11.7 (−12.0 to −11.4)	−5.4 (−5.5 to −5.4)	−8.7 (−9.0 to −8.5)	−3.7 (−3.7 to −3.6)	−2.9 (−3.1 to −2.8)
Estonia§						
Low	−3.2 (−3.4 to −3.1)	−42.8 (−43.7 to −42.0)	−5.7 (−5.9 to −5.4)	−39.0 (−39.4 to −38.6)	−8.9 (−9.3 to −8.4)	−21.2 (−21.5 to −21.0)
High	−4.7 (−4.8 to −4.6)	−27.9 (−28.9 to −26.8)	−7.2 (−7.4 to −7.0)	−22.1 (−23.1 to −21.1)	−10.5 (−11.0 to −9.8)	−11.9 (−12.8 to −11.1)
Lithuania§						
Low	−0.6 (−0.7 to −0.5)	−7.8 (−8.7 to −6.8)	−0.7 (−0.8 to −0.6)	−5.9 (−6.6 to −5.1)	0.3 (0.1 to 0.4)	0.7 (0.2 to 1.2)
High	−2.0 (−2.1 to −2.0)	−12.5 (−13.0 to −11.9)	−2.1 (−2.2 to −2.1)	−8.6 (−9.0 to −8.1)	−1.0 (−1.1 to −0.9)	−1.2 (−1.5 to −1.0)
Women						
Finland						
Low	−4.2 (−4.3 to −4.1)	−12.7 (−12.9 to −12.6)	−5.4 (−5.5 to −5.3)	−8.3 (−8.4 to −8.2)	−4.4 (−4.6 to −4.2)	−3.6 (−3.7 to −3.6)
High	−5.2 (−5.2 to −5.2)	−8.1 (−8.6 to −7.7)	−6.5 (−6.5 to −6.4)	−4.5 (−4.9 to −4.2)	−5.2 (−5.3 to −5.1)	−2.7 (−2.9 to −2.4)
Denmark‡						
Low	−4.0 (−4.1 to −3.9)	−9.6 (−9.7 to −9.4)	−6.5 (−6.7 to −6.4)	−6.3 (−6.4 to −6.1)	−2.9 (−3.0 to −2.8)	−1.9 (−1.9 to −1.8)
High	−5.1 (−5.2 to −5.0)	−6.2 (−6.7 to −5.6)	−8.0 (−8.1 to −7.9)	−3.0 (−3.5 to −2.6)	−4.4 (−4.7 to −4.2)	−1.7 (−2.0 to −1.4)
England and Wales						
Low	−5.6 (−5.8 to −5.3)	−14.8 (−15.0 to −14.6)	−7.0 (−7.4 to −6.5)	−9.4 (−9.6 to −9.2)	−5.8 (−6.3 to −5.1)	−4.0 (−4.2 to −3.9)
High	−6.2 (−6.6 to −5.8)	−10.6 (−12.1 to −9.0)	−7.1 (−7.9 to −6.0)	−4.8 (−6.0 to −3.6)	−8.4 (−9.5 to −6.7)	−4.1 (−5.3 to −3.0)
Austria						
Low	−4.8 (−4.8 to −4.7)	−14.6 (−14.9 to −14.3)	−4.2 (−4.2 to −4.1)	−5.3 (−5.5 to −5.1)	−6.1 (−6.3 to −6.0)	−4.5 (−4.7 to −4.3)
High	−5.2 (−5.6 to −4.9)	−9.1 (−11.1 to −7.0)	−5.9 (−6.5 to −5.5)	−4.1 (−5.6 to −2.7)	−5.2 (−6.4 to −4.6)	−1.8 (−2.7 to −0.9)
Switzerland						
Low	−4.3 (−4.4 to −4.2)	−7.8 (−7.9 to −7.8)	−5.4 (−5.5 to −5.2)	−3.7 (−3.8 to −3.7)	−4.2 (−4.4 to −3.9)	−1.6 (−1.6 to −1.6)
High	−4.5 (−4.6 to −4.5)	−4.4 (−4.8 to −4.0)	−6.4 (−6.6 to −6.1)	−2.1 (−2.4 to −1.8)	−2.6 (−2.9 to −2.4)	−0.6 (−0.8 to −0.4)
Spain (Barcelona)						
Low	−4.4 (−4.6 to −4.2)	−5.6 (−5.7 to −5.5)	−5.3 (−5.7 to −4.8)	−1.9 (−1.9 to −1.8)	−5.2 (−5.6 to −4.7)	−2.0 (−2.0 to −1.9)
High	−3.0 (−3.2 to −2.7)	−2.4 (−2.7 to −2.0)	−4.3 (−5.0 to −3.3)	−0.9 (−1.1 to −0.6)	−5.0 (−5.9 to −3.7)	−1.3 (−1.6 to −1.0)
Italy (Turin)						
Low	−4.7 (−5.0 to −4.4)	−8.5 (−8.7 to −8.4)	−4.6 (−5.1 to −4.1)	−2.4 (−2.5 to −2.3)	−6.0 (−6.5 to −5.3)	−3.3 (−3.4 to −3.2)
High	−6.0 (−6.8 to −4.7)	−6.4 (−7.5 to −5.3)	−7.3 (−9.8 to −0.4)	−2.0 (−2.9 to −1.2)	−6.4 (−8.0 to −3.3)	−2.2 (−3.1 to −1.5)
Italy (Emilia)§						
Low	−5.6 (−5.9 to −5.3)	−6.0 (−6.2 to −5.8)	−7.0 (−7.8 to −6.2)	−2.4 (−2.6 to −2.2)	−5.2 (−5.7 to −4.5)	−1.5 (−1.6 to −1.4)
High	−6.6 (−7.0 to −6.0)	−5.1 (−6.0 to −4.1)	−11.0 (−12.5 to −8.4)	−2.4 (−3.2 to −1.6)	−6.6 (−8.5 to −4.2)	−1.4 (−1.9 to −0.8)
Hungary						
Low	−2.5 (−2.5 to −2.5)	−16.2 (−16.3 to −16.2)	−1.0 (−1.0 to −1.0)	−2.6 (−2.7 to −2.6)	−4.0 (−4.0 to −3.9)	−7.2 (−7.2 to −7.1)
High	−3.1 (−3.1 to −3.0)	−9.9 (−10.6 to −9.3)	−2.3 (−2.4 to −2.2)	−3.3 (−3.7 ot −2.9)	−4.4 (−4.5 to −4.3)	−3.4 (−3.8 to −3.0)
Poland§						
Low	−3.0 (−3.0 to −3.0)	−14.1 (−14.2 to −14.1)	−4.5 (−4.5 to −4.4)	−5.8 (−5.9 to −5.8)	−4.6 (−4.7 to −4.6)	−6.1 (−6.1 to −6.1)
High	−4.2 (−4.3 to −4.1)	−7.8 (−8.3 to −7.4)	−6.6 (−6.7 to −6.5)	−3.5 (−3.8 to −3.3)	−5.9 (−6.0 to −5.7)	−3.0 (−3.3 to −2.8)
Estonia§						
Low	−3.8 (−4.0 to −3.6)	−23.9 (−24.7 to −23.1)	−7.6 (−8.0 to −7.2)	−20.1 (−24.5 to −19.8)	−9.6 (−11.4 to −8.8)	−14.2 (−14.7 to −13.8)
High	−5.2 (−5.3 to −5.1)	−12.8 (−13.5 to −12.1)	−8.5 (−8.8 to −8.1)	−8.3 (−8.9 to −7.7)	−11.8 (−12.4 to −11.1)	−7.8 (−8.5 to −7.1)
Lithuania§						
Low	−1.0 (−1.1 to −0.8)	−6.1 (−6.9 to −5.4)	−0.4 (−0.5 to −0.2)	−1.3 (−1.8 to −0.7)	−1.0 (−1.2 to −0.8)	−1.8 (−2.1 to −1.4)
High	−2.9 (−3.0 to −2.8)	−7.3 (−7.8 to −6.8)	−3.3 (−3.4 to −3.3)	−4.4 (−4.8 to −4.0)	−2.8 (−2.9 to −2.7)	−2.2 (−2.5 to −1.9)

*Average per annum percent change in mortality.

†Average per annum absolute change in mortality (deaths per 100 000 person-years).

‡Average per annum changes between 2010–2014 and 1995–1999.

§Average per annum changes between 2010–2014 and 2000–2004.

### Trends in mortality inequalities by education

Changes in mortality rates among low and high educated caused important changes in the magnitude of inequalities; regardless of the measure, inequalities measured on an absolute scale mostly decreased, while those measured on a relative scale generally rose over time (figure 1, table 2, online supplementary table S6).

**Table 2:** Population attributable risk (PAR; per 100 000 person-year), population attributable fraction (PAF; %), Relative Index of Inequality (RII), Slope Index of Inequality (SII) (95% CI) by educational level for ischaemic and cerebrovascular disease mortality in 1990–1994 (or as otherwise specified) versus 2010–2014, by population and gender

	PAR (100 000 person-years)			PAF (%)		SII (95% CI)		RII (95% CI)	
	1990–1994*		2010–2014	1990–1994*	2010–2014	1990–1994*	2010–2014	1990–1994*	2010–2014
Men									
Ischaemic heart disease									
Finland	205.5 (197.1 to 214.4)		97.0 (95.1 to 99.2)	33.4 (31.7 to 35.2)	41.9 (40.5 to 43.5)	395.9 (365.9 to 425.9)	231.1 (217.5 to 244.7)	2.20 (2.07 to 2.33)	2.96 (2.76 to 3.17)
Denmark	123.3 (116.7 to 130.6)		41.7 (40.3 to 43.2)	36.8 (34.4 to 39.3)	41.8 (39.6 to 44.0)	193.4 (172.1 to 214.8)	97.1 (88.5 to 105.8)	2.03 (1.90 to 2.17)	2.86 (2.60 to 3.15)
England and Wales	175.9 (152.6 to 201.4)		40.2 (32.0 to 49.2)	32.8 (27.4 to 38.4)	25.4 (18.2 to 32.4)	407.1 (299.8 to 514.4)	124.8 (66.3 to 183.3)	2.70 (2.06 to 3.53)	2.31 (1.51 to 3.55)
Austria	121.8 (101.1 to 144.6)		58.4 (54.8 to 62.0)	29.5 (23.6 to 35.5)	34.3 (31.4 to 37.0)	71.4 (27.9 to 114.9)	114.6 (99.0 to 130.1)	1.21 (1.09 to 1.34)	1.92 (1.75 to 2.12)
Switzerland	69.4 (65.3 to 73.6)		32.2 (31.1 to 33.4)	24.6 (22.8 to 26.4)	33.2 (31.4 to 35.1)	139.1 (122.9 to 155.3)	90.0 (80.9 to 99.2)	1.65 (1.55 to 1.75)	2.81 (2.53 to 3.12)
Spain (Barcelona)	36.4 (28.5 to 44.7)		24.3 (20.2 to 28.8)	19.5 (14.6 to 24.3)	28.5 (22.1 to 34.6)	27.1 (-0.3 to 54.5)	38.4 (18.4 to 58.3)	1.19 (1.01 to 1.40)	1.66 (1.28 to 2.15)
Italy (Turin)	40.4 (20.4 to 58.1)		17.7 (6.6 to 29.8)	18.2 (9.9 to 26.6)	19.4 (4.7 to 33.2)	31.9 (-12.9 to 76.7)	41.8 (11.1 to 72.5)	1.26 (1.03 to 1.55)	1.57 (1.08 to 2.28)
Italy (Emilia)	40.3 (30.2 to 51.1)		23.4 (18.8 to 28.2)	26.1 (18.0 to 33.9)	35.5 (25.8 to 44.3)	67.5 (34.8 to 100.2)	46.8 (28.4 to 65.1)	1.60 (1.30 to 2.10)	2.10 (1.50 to 2.90)
Hungary	143.1 (133.3 to 153.6)		198.0 (194.8 to 201.6)	22.5 (20.8 to 24.2)	45.9 (44.7 to 47.2)	141.2 (111.7 to 170.7)	488.7 (470.3 to 507.1)	1.44 (1.38 to 1.51)	3.99 (3.78 to 4.22)
Poland	151.1 (148.0 to 154.3)		114.3 (112.7 to 116.0)	43.1 (41.9 to 44.3)	48.6 (47.6 to 49.7)	323.8 (313.8 to 333.8)	245.9 (240.6 to 251.2)	3.04 (2.94 to 3.15)	3.35 (3.26 to 3.44)
Estonia	300.3 (288.7 to 312.8)		153.9 (148.5 to 159.7)	40.9 (38.7 to 43.2)	44.7 (41.8 to 47.5)	616.6 (569.3 to 663.8)	356.2 (319.4 to 393.0)	2.57 (2.39 to 2.78)	2.98 (2.63 to 3.37)
Lithuania	309.7 (299.9 to 320.7)		283.7 (277.6 to 290.4)	41.2 (39.5 to 43.1)	44.3 (42.7 to 45.9)	531.3 (497.4 to 565.1)	542.3 (509.6 to 575.0)	2.24 (2.12 to 2.37)	2.63 (2.46 to 2.80)
Cerebrovascular disease									
Finland	40.1 (34.6 to 45.3)		23.6 (22.2 to 25.0)	23.7 (20.0 to 27.2)	34.2 (30.9 to 36.9)	72.1 (55.1 to 89.1)	54.6 (46.9 to 62.3)	1.74 (1.56 to 1.94)	2.38 (2.10 to 2.70)
Denmark	31.8 (26.8 to 36.7)		18.3 (16.9 to 19.7)	28.0 (22.9 to 32.8)	32.6 (29.1 to 35.8)	40.1 (27.0 to 53.2)	46.1 (39.4 to 52.7)	1.62 (1.44 to 1.82)	2.52 (2.22 to 2.87)
England and Wales	36.4 (23.3 to 50.1)		8.8 (2.8 to 15.0)	28.3 (15.4 to 40.0)	24.3 (3.5 to 41.7)	81.6 (25.8 to 137.5)	24.0 (-4.7 to 52.8)	2.20 (1.27 to 3.82)	2.20 (0.87 to 5.55)
Austria	53.1 (39.8 to 66.0)		16.1 (14.4 to 18.1)	32.8 (23.1 to 41.6)	34.8 (29.7 to 40.5)	104.2 (77.0 to 131.3)	35.9 (27.9 to 43.9)	1.90 (1.60 to 2.25)	2.22 (1.84 to 2.67)
Switzerland	20.9 (18.6 to 23.1)		8.1 (7.4 to 8.8)	26.7 (23.0 to 30.1)	30.0 (25.7 to 33.6)	49.6 (41.1 to 58.1)	20.6 (15.8 to 25.5)	1.97 (1.75 to 2.21)	2.42 (1.98 to 2.95)
Spain (Barcelona)	27.9 (21.9 to 34.5)		8.4 (5.2 to 11.7)	26.3 (19.4 to 33.3)	22.6 (12.0 to 32.4)	46.2 (25.8 to 66.6)	16.9 (3.8 to 30.0)	1.72 (1.36 to 2.17)	1.68 (1.13 to 2.49)
Italy (Turin)	44.9 (33.3 to 56.1)		8.1 (-0.4 to 16.6)	33.3 (22.7 to 42.8)	17.3 (-4.7 to 35.5)	90.7 (58.7 to 122.7)	10.5 (-12.1 to 33.1)	2.08 (1.54 to 2.80)	1.18 (0.71 to 1.98)
Italy (Emilia)	2.5 (-5.5 to 10.9)		5.3 (1.7 to 9.8)	4.6 (-11.6 to 19.6)	19.0 (2.3 to 35.8)	17.0 (-2.8 to 36.9)	12.8 (0.6 to 25.1)	1.40 (1.00 to 2.20)	1.70 (1.10 to 2.80)
Hungary	197.7 (191.9 to 203.6)		99.2 (97.6 to 100.9)	51.7 (49.6 to 53.7)	58.6 (56.7 to 60.5)	365.7 (345.9 to 385.6)	247.4 (236.4 to 258.4)	3.37 (3.14 to 3.63)	6.81 (6.18 to 7.50)
Poland	111.2 (109.2 to 113.3)		75.2 (74.0 to 76.6)	54.6 (53.1 to 56.1)	53.2 (51.8 to 54.8)	242.1 (234.7 to 249.4)	172.5 (168.4 to 176.5)	4.29 (4.08 to 4.51)	4.34 (4.18 to 4.50)
Estonia	112.4 (103.9 to 121.5)		39.1 (35.5 to 42.2)	37.8 (33.9 to 41.7)	41.8 (35.5 to 47.0)	236.5 (206.0 to 267.1)	92.6 (73.4 to 111.7)	2.51 (2.23 to 2.82)	3.00 (2.36 to 3.80)
Lithuania	103.1 (97.4 to 109.0)		96.4 (92.6 to 100.2)	44.6 (41.2 to 48.0)	45.5 (42.6 to 48.2)	161.7 (143.0 to 180.4)	181.5 (162.9 to 200.1)	2.21 (1.99 to 2.44)	2.66 (2.38 to 2.97)
Women									
Ischaemic heart disease									
Finland	103.3 (97.7 to 109.2)		30.2 (28.9 to 31.6)	46.2 (43.1 to 49.5)	47.2 (44.0 to 50.4)	184.1 (168.1 to 200.0)	70.7 (63.7 to 77.7)	2.76 (2.50 to 3.06)	3.76 (3.26 to 4.34)
Denmark	72.3 (67.4 to 77.5)		19.3 (18.4 to 20.2)	53.0 (48.4 to 57.6)	51.1 (47.3 to 54.9)	127.5 (114.3 to 140.6)	47.3 (42.4 to 52.1)	3.83 (3.36 to 4.37)	4.92 (4.14 to 5.85)
England and Wales	106.8 (86.8 to 127.6)		22.9 (17.7 to 29.1)	47.6 (37.0 to 58.2)	42.1 (27.8 to 55.5)	228.1 (155.6 to 300.6)	63.0 (30.8 to 95.1)	4.87 (2.72 to 8.72)	4.37 (1.83 to 10.44)
Austria	53.3 (27.6 to 81.9)		30.0 (25.2 to 35.4)	31.2 (15.1 to 48.2)	46.3 (37.5 to 55.6)	87.7 (63.8 to 111.6)	55.1 (45.9 to 64.3)	1.72 (1.47 to 2.01)	2.52 (2.15 to 2.96)
Switzerland	37.4 (31.4 to 43.6)		13.5 (11.8 to 15.2)	39.9 (32.6 to 47.2)	45.6 (38.3 to 52.9)	72.2 (63.7 to 80.7)	28.4 (23.8 to 33.1)	2.33 (2.10 to 2.59)	3.07 (2.55 to 3.70)
Spain (Barcelona)	25.9 (20.6 to 31.7)		5.9 (3.2 to 8.7)	47.6 (35.5 to 59.9)	31.1 (13.5 to 47.3)	43.3 (29.0 to 57.7)	13.1 (4.2 to 22.0)	2.76 (1.81 to 4.22)	2.39 (1.27 to 4.51)
Italy (Turin)	23.3 (9.0 to 39.6)		15.5 (7.9 to 25.1)	31.9 (9.9 to 55.1)	55.4 (23.9 to 90.9)	30.9 (3.6 to 58.1)	30.6 (15.2 to 46.0)	1.64 (1.06 to 2.55)	3.53 (1.53 to 8.14)
Italy (Emilia)	10.3 (2.3 to 19.8)		9.7 (6.9 to 12.8)	22.7 (2.8 to 43.8)	46.9 (28.2 to 64.5)	16.6 (-1.2 to 34.3)	11.7 (1.6 to 21.8)	1.90 (1.21 to 3.72)	1.70 (1.00 to 3.10)
Hungary	109.1 (98.9 to 120.0)		86.9 (82.6 to 91.0)	37.7 (33.8 to 41.8)	43.9 (41.2 to 46.4)	96.4 (72.9 to 119.9)	218.6 (207.6 to 229.7)	1.69 (1.55 to 1.84)	4.52 (4.18 to 4.89)
Poland	66.0 (62.9 to 69.3)		45.0 (43.8 to 46.3)	48.8 (46.1 to 51.6)	54.7 (52.8 to 56.7)	132.5 (126.8 to 138.1)	99.3 (96.6 to 101.9)	3.42 (3.23 to 3.62)	4.41 (4.22 to 4.61)
Estonia	139.5 (132.6 to 147.3)		47.1 (44.3 to 50.0)	48.4 (45.5 to 51.2)	46.0 (41.5 to 50.1)	287.3 (265.0 to 309.6)	117.7 (101.9 to 133.5)	3.40 (3.07 to 3.78)	3.71 (3.06 to 4.49)
Lithuania	144.1 (137.3 to 150.8)		120.8 (117.5 to 124.1)	48.4 (45.5 to 52.3)	52.5 (50.3 to 54.8)	236.0 (218.6 to 253.4)	250.1 (235.0 to 265.1)	2.67 (2.47 to 2.90)	3.92 (3.57 to 4.29)
Cerebrovascular disease									
Finland	34.0 (29.0 to 39.3)		12.3 (11.0 to 13.7)	30.1 (25.0 to 35.2)	30.1 (47.4 to 57.4)	65.1 (53.1 to 77.1)	32.9 (27.0 to 38.9)	1.97 (1.74 to 2.25)	2.50 (2.11 to 2.96)
Denmark	27.2 (22.9 to 32.1)		12.9 (11.6 to 14.4)	34.7 (28.2 to 41.7)	33.3 (28.8 to 37.9)	41.9 (31.1 to 52.6)	34.8 (29.6 to 40.1)	2.08 (1.78 to 2.43)	3.03 (2.58 to 3.56)
England and Wales	15.8 (-3.0 to 36.6)		14.2 (10.0 to 19.0)	14.3 (-4.5 to 33.0)	42.1 (22.4 to 59.4)	32.7 (-29.6 to 95.0)	36.5 (11.5 to 61.4)	1.61 (0.84 to 3.07)	3.67 (1.24 to 10.84)
Austria	57.2 (41.3 to 75.2)		11.2 (7.4 to 15.2)	50.9 (34.8 to 68.3)	37.0 (22.2 to 51.3)	90.2 (71.6 to 108.7)	22.5 (16.2 to 28.8)	2.44 (1.99 to 2.99)	2.20 (1.75 to 2.77)
Switzerland	17.9 (12.9 to 22.4)		3.0 (1.0 to 5.2)	37.2 (25.4 to 47.2)	14.2 (3.9 to 24.2)	31.1 (24.9 to 37.3)	9.7 (5.5 to 13.9)	1.99 (1.72 to 2.30)	1.61 (1.31 to 1.99)
Spain (Barcelona)	21.4 (14.7 to 27.6)		6.2 (2.8 to 10.3)	35.5 (22.2 to 46.6)	28.5 (9.7 to 47.8)	32.9 (16.8 to 49.0)	11.9 (1.9 to 21.9)	1.89 (1.30 to 2.74)	1.74 (1.00 to 3.03)
Italy (Turin)	27.7 (11.0 to 45.7)		9.9 (2.6 to 19.0)	32.3 (10.7 to 53.9)	35.9 (3.6 to 69.0)	40.5 (10.5 to 70.5)	17.2 (1.2 to 33.3)	1.76 (1.16 to 2.68)	1.95 (0.91 to 4.18)
Italy (Emilia)	6.6 (−0.6 to 14.4)		4.2 (0.0 to 8.6)	19.3 (−4.6 to 42.0)	23.2 (−4.9 to 47.4)	15.7 (0.5 to 30.9)	13.0 (3.5 to 22.5)	1.60 (1.00 to 2.70)	2.30 (1.20 to 4.50)
Hungary	135.0 (126.1 to 143.2)		45.8 (43.4 to 48.7)	53.5 (49.4 to 57.2)	50.1 (46.5 to 54.1)	203.4 (184.4 to 222.5)	110.2 (102.9 to 117.4)	2.82 (2.55 to 3.13)	5.15 (4.57 to 5.80)
Poland	71.8 (68.7 to 74.7)		45.2 (43.9 to 46.4)	52.6 (49.9 to 55.1)	54.7 (52.6 to 56.7)	147.5 (142.0 to 152.9)	99.6 (97.0 to 102.3)	3.83 (3.62 to 4.06)	4.27 (4.09 to 4.46)
Estonia	66.4 (58.8 to 73.5)		19.0 (16.9 to 21.3)	36.7 (31.5 to 41.5)	39.9 (33.1 to 46.1)	151.6 (133.0 to 170.2)	52.3 (41.0 to 63.6)	2.67 (2.35 to 3.03)	3.50 (2.63 to 4.64)
Lithuania	66.1 (60.7 to 71.7)		53.9 (51.0 to 56.9)	42.2 (37.9 to 46.5)	44.2 (40.8 to 47.6)	113.4 (100.3 to 126.4)	111.3 (99.8 to 122.8)	2.39 (2.14 to 2.66)	3.03 (2.68 to 3.43)

* 1995–1999 for Denmark and 2000–2004 for Italy (Emilia), Poland, Estonia and Lithuania.

In both genders, absolute inequalities in total cardiovascular, IHD and CVD mortality, measured through the SII, went markedly down in most populations or remained stable. Nonetheless, such favourable trend was not consistently seen in Hungary (total cardiovascular mortality among men, IHD mortality in both genders) where the SII noticeably increased by the 2010s. The PAR of all groups of causes mirrored the SII patterns and decreased in almost all populations.

Irrespective of gender, relative inequalities in total cardiovascular mortality, measured through the RII, increased in most countries but remained fairly stable over time in England and Wales, Southern European populations and Estonia. Changes in relative inequalities in IHD mortality showed a very similar picture; relative inequalities in CVD mortality did not uniformly rise and, among men in Turin (Italy), the less educated caught up and differences became non-significant. The PAF of all groups of causes rose almost uniformly among men; among women patterns were muddled.

**Figure 2:**
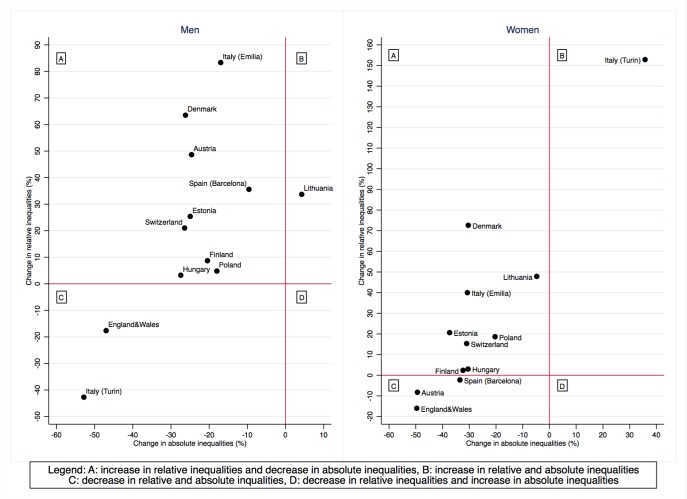
Changes in absolute and relative educational inequalities in total cardiovascular disease mortality between 2000-2004 (period in which data became available for all populations) and 2010–2014, by population and gender, 35–79 years. Note: changes in relative inequalities (ratio measures) were calculated using the following formula where RII stands for Relative Index of Inequality: 100*(RII_2010-14_-RII_2000-__04_)/(RII_2000-04_ – 1). Changes in absolute inequalities (difference measures) were calculated using the following formula where SII stands for Slope Index of Inequality: 100* SII_2010-14_-SII_2000-04_)/(SII_2000-04_).

Figure 2 presents a summary picture of changes in absolute and relative inequalities in total cardiovascular mortality between the 2000s and the 2010s. Being in the upper left hand quadrant of this graph means that absolute inequalities declined over time while relative inequalities increased, while being in the lower left hand quadrant implies that both absolute and relative inequalities declined. The latter was the case for both genders in England and Wales, women in Austria and Barcelona (Spain) and men in Turin (Italy). A country’s position in the upper right hand quadrant implies instead an increase in absolute and relative inequalities; this happened among men in Lithuania and women in Turin (Italy). Summary graphs for IHD and CVD mortality showed similar patterns (online supplementary figures S3–S4). Of note, a decrease in absolute inequalities coupled with a decrease or a stable trend in relative inequalities for all groups of causes was seen among men in England and Wales and in Turin (Italy).

### Current educational differences between countries

**Figure 3:**
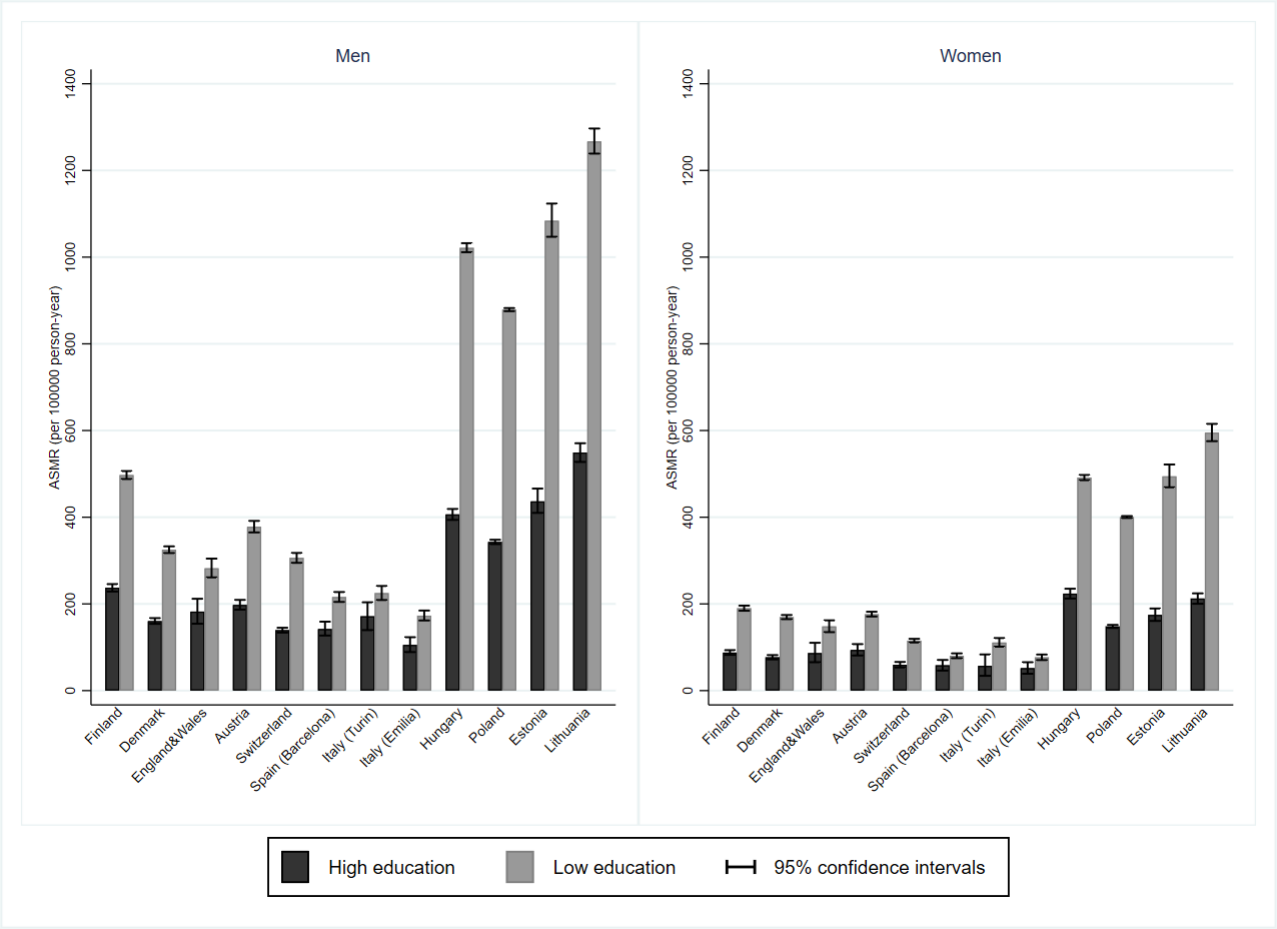
Total cardiovascular disease age-standardised mortality rates (ASMR) and 95% CI among low and high educated, by population and gender, 35-79 years, 2010–2014.

In the 2010s, age-standardised cardiovascular mortality rates were higher among low than high educated men and women in all European populations, but the absolute gap was very much larger in Central-Eastern European and Baltic countries than elsewhere, especially in Southern Europe (Barcelona: Spain; Turin and Emilia: Italy) (figure 3). Moreover, a threefold geographic divide was apparent, especially among men, whereby educational inequalities in cardiovascular mortality were lowest in Southern European populations, of intermediate magnitude in Nordic and Western countries and highest in Baltic and Central-Eastern European countries. Irrespective of the measure, Southern European populations and, to some extent, Austria and Switzerland, combined small absolute with small relative inequalities; conversely, Central-Eastern European countries combined large absolute with large relative inequalities.

### Trends and inequalities by occupation

For those populations in which an assessment of occupational inequalities in cardiovascular mortality was possible, the picture was largely similar to that seen for educational inequalities (online supplementary figure S5, table S7–S11). Manual workers, who had the highest burden of death, experienced the largest absolute declines in mortality, whereas men in upper non-manual jobs, who had the lowest mortality, experienced the largest relative declines. Turin (Italy) and Lithuania stood out because both absolute and relative declines were larger among the manual workers in the former and among the upper non-manual employees in the latter. Also for occupational differences, the absolute gap generally narrowed while the relative gap widened and by the 2010s a geographical divide was appreciable.

## Discussion

This study provides a robust and up-to-date analysis of trends in relative and absolute socioeconomic inequality in cardiovascular disease mortality in Europe. It covered two-and-a-half decades and 12 populations, allowing to appreciate how inequalities have evolved over time and geographically. Moreover, for men in most of the populations included in the comparison, two different aspects of socioeconomic stratification were measured, and because the overall results for education and occupation were similar, this considerably strengthens the conclusions.

The analysis of cardiovascular mortality rate trends showed that lower socioeconomic groups experienced strong declines; this suggests that the benefits of cardiovascular prevention and treatment have reached all socioeconomic groups. These declining trends are likely to reflect favourable changes in either health-related behaviours (eg, smoking, diet and alcohol consumption) or healthcare effectiveness (eg, hypertension detection and treatment, statin prescriptions and thrombolytic therapy) or both. Few studies have estimated the quantitative contribution of these factors to trends in inequalities. In Denmark, declines in IHD mortality between 1991 and 2007 came from favourable changes in risk factors shared among all socioeconomic groups.^12^ In England and Scotland instead the narrowing of absolute inequalities in the 2000s was attributed to a socially uniform uptake of treatments rather than risk factor changes.^13 14^ An interpretation in terms of reasonably equal access and quality of medical care in the decline of cardiovascular mortality in Europe is supported by a previous study of trends in mortality from conditions amenable to medical interventions, which also showed a considerable reduction in mortality from these conditions among the low educated and a narrowing of absolute inequalities.^15^

The results of our study showed that absolute inequalities in cardiovascular mortality have often narrowed but relative inequalities mostly widened, confirming previous evidence.^6 16^ Researchers and policymakers do not agree on what measures to use for monitoring progress in tackling health inequalities, particularly on whether to use relative or absolute measures. Focusing on relative or absolute measures of inequalities ultimately depends on one’s normative standpoint.^17^ Using relative measures implies an egalitarian position, in which what matters is equality in itself, independent of other considerations such as the absolute disease rates for each group. Using absolute measures implies the pragmatic view that absolute rates matter most for people in lower socioeconomic groups and that a smaller absolute mortality excess is still important even if it goes together with a larger relative mortality excess.^18^ Therefore, from a pragmatic view, the narrowing of absolute inequalities in cardiovascular mortality is a very favourable development, despite the concurrent rise in relative inequalities, which as scenario calculations have shown, may be almost inevitable in a context of overall declining cardiovascular disease mortality.^19^

There were also instances in which both absolute and relative inequalities narrowed over time. This happened in England and Wales where, between 1997 and 2010, an ambitious intersectorial national programme was carried out to tackle health inequalities.^20^ Recent evidence showed that this programme may have reduced geographical inequalities in life expectancy.^21^ Moreover, since the mid-2000s, the Department of Health launched a series of actions aimed at narrowing cardiovascular disease inequalities, including a lifestyle counselling and active treatment offer, that contributed to population-wide reductions in smoking levels and increases in statins use, and a screening programme, whose coverage did not differ by deprivation.^22^ In Turin (Italy), socioeconomic disparities in CVD mortality declined substantially among men; no such notable reduction was seen for IHD inequalities, which indicates the presence of CVD-specific factors, for example, alcohol consumption. The decline in educational disparities in alcohol-related mortality in Turin over time coupled with the finding of higher alcohol consumption among high educated men^23^ points to the role of changing patterns of alcohol intake in narrowing CVD inequalities.

This study adds to the evidence on geographic differences and confirms that countries differ enormously in the burden and in the magnitude of cardiovascular mortality inequalities with Southern Europe having the smallest rates and inequalities and Central-Eastern and Baltic countries having the largest ones. The socially widespread adoption of the Mediterranean diet, which is associated with a lower risk of cardiovascular disease, is a potential explanation for the comparatively low cardiovascular mortality rates and socioeconomic differences in Southern Europe.^24^ However, a recent Italian study reported an association between the economic recession and lower adherence to the Mediterranean diet among the most deprived groups,^25^ suggesting that the levelling effect of the Mediterranean diet on cardiovascular mortality inequalities may be lost in the future.

Larger inequalities in Central-Eastern European and Baltic countries have been reported for cardiovascular mortality and for other causes of deaths and attributed to a combination of inequalities in smoking, excessive alcohol consumption and lack of access to good quality healthcare, against a background of the transition to a market economy in the 1990s.^26^

### Limitations

Despite a careful data harmonisation, data comparability issues may still affect the results. Potential sources of bias are the variations over time and between countries in certification and coding of causes of death. The miscoding of IHD may threaten the validity of cross-countries comparisons.^27^ To evaluate the potential for misclassification, we performed a sensitivity analysis and re-estimated absolute and relative inequalities by education for IHD combined with other heart disease (online supplementary figure S6). The picture did not change substantially, and although a certain degree of misclassification cannot be ruled out, it appears unlikely that this alone can explain our findings. The coding of CVD deaths is similarly critical. In Estonia, changes in coding practices resulted in a sharp decrease in cerebrovascular mortality since the 2000s.^28^ These changes, although likely not sensitive to SEP, may still affect absolute inequalities, and therefore, figures for Estonia must be interpreted cautiously.

Differences in population coverage exist between countries included in the study. For Spain, data came from Barcelona only. A longitudinal mortality study based on the 2001 census compared educational inequalities between the entire country and subnational areas and concluded that Barcelona does not misrepresent the country.^29^ For Italy, data came from Turin and Emilia, two Northern urban and wealthy areas. A nationwide cohort study based on the 2011 census showed that the educational gap in cardiovascular mortality was slightly wider among men in the North and women in the South^30^ suggesting that including only cohorts from Northern areas may overestimate among males and underestimate among females the magnitude of inequalities for the whole country.

In England and Wales, low and middle levels of education could not be distinguished. A wider gradient could have been concealed by the lack of a more precise stratification, and therefore, inequalities’ estimates should be cautiously interpreted. Nonetheless, the consistency with the results by occupational class provides additional evidence for a decrease in absolute inequalities and a substantial stability in relative inequalities.

## Conclusions

Lower socioeconomic groups have experienced remarkable declines in cardiovascular mortality rates over the last 25 years, and trends in inequalities can be qualified as favourable overall. Nevertheless, further reducing inequalities in cardiovascular mortality, especially in the Nordic, Central-European and Baltic countries, remains an important challenge for European health systems and policies.

**Key messages**

**What is already known on this subject?**

- Cardiovascular diseases are still a leading cause of death in Europe, although death rates decreased remarkably over the last three decades.
- Deaths from cardiovascular disease are inequitably distributed across socioeconomic groups, and larger relative declines in cardiovascular mortality have been observed among individuals in higher socioeconomic positions in the 1980s and 1990s in many European countries.
- Less attention has been paid to what happened to absolute inequalities, which express the difference between the mortality rates of the highest and the lowest socioeconomic groups.

**What might this study add?**

- We systematically assessed both ratio measures of relative inequalities and difference measure of absolute inequalities in cardiovascular mortality and showed that, although relative inequalities increased over time, absolute inequalities often declined substantially on all measures used in the 12 European populations analysed.
- In most of the populations, two different aspects of the socioeconomic position, educational level and occupational class, were analysed for men, and because the overall results for education and occupation are similar, it considerably strengthens our conclusions.

**How might this impact on the clinical practice?**

- Equity-oriented healthcare services with an appropriate emphasis on both prevention and treatment have the potential to further reduce the gap in cardiovascular disease mortality and thereby the gap in total mortality between socioeconomic groups, which is still substantial in most countries.
- Studying how England and Wales, the best performer country in this study, has achieved a large reduction of absolute inequalities in cardiovascular disease mortality without increasing relative inequalities may provide lesson clues for other countries’ health systems.
