# Supplementary material for: Progress in reducing inequalities in cardiovascular disease mortality in Europe
Source: Heart. 2019 Aug 22;106(1):40–9. doi: 10.1136/heartjnl-2019-315129 (PMC6952836; doi:10.1136/heartjnl-2019-315129)
Supplement: Supplementary data [file heartjnl-2019-315129supp001.pdf]

List of Supplementary Tables

Supplementary Table S 1. Overview and characteristics of data sources 2
Supplementary Table S 2. List of the ICD codes of the causes of death 3
Supplementary Table S 2. Distribution of person-years (py) and cardiovascular disease (CVD), ischaemic heart disease (IHD) and cerebrovascular disease (CeVD) deaths by educational level, period and population, men, 35-79 years 4
Supplementary Table S 3. Distribution of person-years (py) and cardiovascular disease (CVD), ischaemic heart disease (IHD) and cerebrovascular disease (CeVD) deaths by educational level, period and population, women, 35-79 years 5
Supplementary Table S 4. Distribution of person-years (py) and cardiovascular disease (CVD), ischeamic heart disease (IHD) and cerebrovascular disease (CeVD) deaths by occupational class, period and population, men, 35-64 years 6
Supplementary Table S 6.Population-Attributable Risk (PAR, per 100,000 person-year), Population-Attributable Fraction (PAF, %), Relative Index of Inequality (RII), Slope Index of Inequality (SII) with 95% confidence intervals (CI) of education for cardiovascular disease mortality between beginning of observation periods and 2010-14. 7
Supplementary Table S 7. Age-standardised cardiovascular disease mortality rates per 100,000 person years, by occupational class, period and population, men, 35-64 years 8
Supplementary Table S 8. Age-standardised ischaemic heart disease mortality rates per 100,000 person years, by occupational class, period and population, men, 35-64 years 9
Supplementary Table S 9. Age-standardised cerebrovascular disease mortality rates per 100,000 person years, by occupational class, period and population, men, 35-64 years 10
Supplementary Table S 10. Relative and absolute annualised changes in age-standardised mortality rates between end and beginning of observation periods for cardiovascular, ischaemic and cerebrovascular disease mortality, by upper non-manual and manual occupation, men, 35-64 years 11
Supplementary Table S 11. Population-Attributable Risk (PAR, per 100,000 person-year) and Population-Attributable Fraction (PAF, %) of occupational class for cardiovascular disease mortality, ischaemic heart disease mortality and cerebrovascular disease mortality between beginning of observation periods and 2010-14 12

List of Supplementary Figures

Supplementary Figure S 1.Trend in ischaemic heart disease mortality (ASMR: age-standardised mortality rates) and inequalities (Relative and Slope Index of Inequality) in mortality by educational level, population and gender, 35-79 years 13
Supplementary Figure S 2. Trend in cerebrovascular disease mortality (ASMR: age-standardised mortality rates) and inequalities (Relative and Slope Index of Inequality) in mortality by educational level, population and gender, 35-79 years 14
Supplementary Figure S 3. Changes in absolute and relative educational inequalities in ischaemic heart disease mortality between 2000-04 and 2010-14, by population and gender, 35-79 years 15
Supplementary Figure S 4. Changes in absolute and relative educational inequalities in cerebrovascular disease mortality between 2000-04 and 2010-14, by population and gender, 35-79 years 15
Supplementary Figure S 5. Trends in cardiovascular disease mortality (ASMR: age-standardised mortality rates) and inequalities by occupation class, men, 35-64 years 16
Supplementary Figure S 6. Trends in Relative Index of Inequality and Slope Index of Inequality for ischaemic heart disease and other heart diseases mortality by education 18

Di Girolamo C, *et al. Heart* 2019; 106:40–49. doi: 10.1136/heartjnl-2019-315129

Supplementary Table S 1. Overview and characteristics of data sources

Population	Design	Observation periods (census year)<br>1990-1994	1995-1999	2000-2004	2005-2009	2010-2014	Geographic coverage	Population coverage	Occupational class availability	Educational level availability
Nordic countries										
**Finland**	Longitudinal	1990-1995<br>(1990)	1995-2000	2000-2005<br>(2000)	2005-2010	2010-2014*<br>(2010)	National	Whole population (*80% representative sample)	upper non-manual, lower non-manual, manual, farmers, self-employed	Low (ISCED 0,1,2), mid (ISCED 3,4), high (ISCED 5,6)
**Denmark**	Longitudinal	na<br>na	1995-1999<br>(1995)	2000-2004<br>(2000)	2005-2009<br>(2005)	2010-2014<br>(2010)	National	Whole population	upper non-manual, lower non-manual, manual, farmers, self-employed	Low (ISCED 0,1,2), mid (ISCED 3,4), high (ISCED 5,6)
Western countries										
**England and Wales**	Longitudinal	1991-1996<br>(1991)	1996-2001	2001-2006<br>(2001)	2006-2011	2011-2013<br>(2011)	National	1% representative sample	upper non-manual, lower non-manual, manual, farmers (not available), self-employed (only 2001-2011)	Low and mid (ISCED 0-4), high (ISCED 5,6)
**Austria**	Longitudinal	1991-1992<br>(1991)		2001-2002<br>(2001)		2011-2013<br>(2011)	National	Whole population	non-manual (upper and lower combined), manual, farmers (not available in 2011), self-employed	Low (ISCED 0,1,2), mid (ISCED 3,4), high (ISCED 5,6)
**Switzerland**	Longitudinal	1990-1995<br>(1990)	1995-2000	2000-2005<br>(2000)	2005-2010	2010-2014<br>(2010)	National	Swiss nationals	upper non-manual, lower non-manual, manual, farmers, self-employed	Low (ISCED 0,1,2), mid (ISCED 3,4), high (ISCED 5,6)
Southern countries										
**Spain, Barcelona**	Repeated cross-sectional	1992-1996	1997-2001	2002-2006	2007-2010	2010-2014	City	Whole population	Not available	Low (ISCED 0,1,2), mid (ISCED 3,4), high (ISCED 5,6)
**Italy, Turin**	Longitudinal	1991-1996<br>(1991)	1996-2001	2001-2006<br>(2001)	2006-2011	2011-2013<br>(2011)	City	Whole population	upper non-manual, lower non-manual, manual, farmers (not available in 1991), self-employed; occ class not available in 2011	Low (ISCED 0,1,2), mid (ISCED 3,4), high (ISCED 5,6)
**Italy, Emilia**	Longitudinal	na<br>na	na	2001-2006<br>(2001)	2006-2011	2011-2016<br>(2011)	Three cities (Bologna, Modena, Reggio Emilia)	Whole population	Not available	Low (ISCED 0,1,2), mid (ISCED 3,4), high (ISCED 5,6)
Central-Eastern countries										
**Hungary**	Cross-sectional	1988-1991<br>(1990)	na	1999-2002<br>(2001)	na	2010-2012<br>(2011)	National	Whole population	Not available	Low (ISCED 0,1,2), mid (ISCED 3,4), high (ISCED 5,6)
**Poland**	Cross-sectional	na<br>na	na	2001-2003<br>(2002)	na	2010-2012<br>(2011)	National	Whole population	Not available	Low (ISCED 0,1,2), mid (ISCED 3,4), high (ISCED 5,6)
Baltic countries										
**Estonia**	Longitudinal	na<br>na	na	2000-2005<br>(2001)	2006-2011	2012-2015<br>(2011)	National	Whole population	upper non-manual, lower non-manual, manual, farmers	Low (ISCED 0,1,2), mid (ISCED 3,4), high (ISCED 5,6)
**Lithuania**	Longitudinal	na<br>na	na	2001-2005<br>(2001)	2006-2009	2011-2014<br>(2011)	National	Whole population	upper non-manual, lower non-manual, manual, farmers, self-employed	Low (ISCED 0,1,2), mid (ISCED 3,4), high (ISCED 5,6)

ISCED: International standard classification of education

Di Girolamo C, *et al. Heart* 2019; 106:40–49. doi: 10.1136/heartjnl-2019-315129

Supplementary Table S 2. List of the ICD codes of the causes of death

	ICD 8 codes	ICD 9 codes	ICD 10 codes
**Cardiovascular disease**	390-458	390-459	I00-I99
**Ischaemic heart disease**	410-414	410-414	I20-I25
**Cerebrovascular disease**	430-438	430-438	I60-I69
**Other heart disease**	420-429	415-417; 420-429	I26-I52; I98

Di Girolamo C, *et al. Heart* 2019; 106:40–49. doi: 10.1136/heartjnl-2019-315129

Supplementary Table S 3. Distribution of person-years (py) and cardiovascular disease (CVD), ischaemic heart disease (IHD) and cerebrovascular disease (CeVD) deaths by educational level, period and population, men, 35-79 years

		1990-1994				1995-1999				2000-2004				2005-2009				2010-2014			
		**py**	**CVD**	**IHD**	**CeVD**	**py**	**CVD**	**IHD**	**CeVD**	**py**	**CVD**	**IHD**	**CeVD**	**py**	**CVD**	**IHD**	**CeVD**	**py**	**CVD**	**IHD**	**CeVD**
Finland	Low	3063862	30020	20488	5260	2738419	24895	16756	4326	2582030	21077	13764	3718	2225260	17397	10947	2953	1622578	11202	6762	1941
	Middle	1700099	5121	3330	941	1851423	5542	3607	968	2432850	6181	3879	1057	2501196	7289	4319	1129	2305363	6487	3703	1050
	High	1300251	3665	2384	705	1364150	3491	2259	648	1788264	3543	2140	691	1830589	3806	2228	664	1787056	3216	1808	579
Denmark	Low	-	-	-	-	2115048	12679	7243	2230	2107680	12668	6072	2672	1968746	8884	3859	1946	1829193	6630	2685	1495
	Middle	-	-	-	-	2744305	8387	4677	1519	3104266	9182	4308	1925	3316392	7754	3292	1791	3462344	6962	2891	1516
	High	-	-	-	-	1255152	2478	1321	487	1502674	2870	1239	679	1734890	2476	966	581	1964224	2276	845	514
England and Wales	Low	470395	3581	2449	558	453846	3006	1951	504	468847	2170	1348	395	445351	1639	999	264	229763	611	390	85
	Middle	-	-	-	-	-	-	-	-	-	-	-	-	-	-	-	-	-	-	-	-
	High	111191	355	234	56	115782	325	214	54	131313	263	176	41	134084	233	131	38	120465	163	107	23
Austria	Low	525388	4389	2170	954	-	-	-	-	441786	3234	1652	597	-	-	-	-	746441	3421	1810	551
	Middle	1063871	5527	3042	986	-	-	-	-	1400269	4903	2780	757	-	-	-	-	2864726	6915	3991	984
	High	130090	436	245	87	-	-	-		195742	399	206	69	-	-	-	-	903567	1285	726	195
Switzerland	Low	1283900	11453	5666	1758	1078287	8622	4441	1309	1043983	5894	2930	891	891006	4477	2209	595	757055	2893	1346	360
	Middle	3523059	14195	7843	1916	3480714	12565	6992	1676	3623813	8641	4543	1190	3557759	8168	4200	1025	3191676	6052	2902	765
	High	1528247	3871	2113	529	1553571	3642	2033	517	2145411	3199	1706	432	2165592	3064	1594	404	1858163	2369	1111	308
Spain, Barcelona	Low	1237926	5304	2319	1348	1100921	4903	2222	1146	1050171	3899	1634	874	792854	2361	960	491	565691	1379	551	264
	Middle	350373	899	421	185	391459	943	448	190	498351	836	359	163	440474	615	250	121	351670	469	216	77
	High	351099	739	347	169	382422	772	353	152	458786	713	322	122	422162	506	205	103	361180	336	142	67
Italy, Turin	Low	802403	4236	1762	1044	678424	3553	1457	812	661870	2810	1206	643	536306	2125	920	517	254954	759	325	174
	Middle	244121	706	323	134	230623	611	258	130	326521	652	275	139	300286	528	232	115	185925	295	124	65
	High	122230	357	148	73	111935	286	112	57	163619	297	133	61	150338	239	107	61	95757	116	50	25
Italy, Emilia	Low	-	-	-	-	-	-	-	-	465378	2069	1052	373	375878	1408	655	274	387631	947	412	180
	Middle	-	-	-	-	-	-	-	-	262980	443	236	69	247752	367	167	73	352386	341	168	62
	High	-	-	-	-	-	-	-	-	157449	257	121	52	147061	187	87	35	215919	162	66	33
Hungary	Low	6865976	88657	39480	24367	-	-	-	-	6221876	76848	35582	20655	-	-	-	-	4448391	38777	20249	8244
	Middle	1469996	9982	5548	2175	-	-	-	-	1960936	10326	5391	2391	-	-	-	-	1865685	7482	4145	1324
	High	1139288	6127	3551	1229	-	-	-	-	1363220	5881	3167	1364	-	-	-	-	1308606	4279	2465	721
Poland	Low	-	-	-	-	-	-	-	-	15636906	139782	53409	30957	-	-	-	-	15147009	115255	37521	22605
	Middle	-	-	-	-	-	-	-	-	7092219	27692	12001	5758	-	-	-	-	7752717	29308	10423	5423
	High	-	-	-	-	-	-	-	-	2875902	9000	4105	1810	-	-	-	-	3922338	9484	3393	1789
Estonia	Low	-	-	-	-	-	-	-	-	498889	10120	5962	2426	378745	7620	3907	1260	242230	3352	1481	413
	Middle	-	-	-	-	-	-	-	-	911196	5801	3258	1287	912855	6091	2914	984	710737	3684	1642	422
	High	-	-	-	-	-	-	-	-	297283	1624	942	389	291064	1556	810	245	270683	995	433	122
Lithuania	Low	-	-	-	-	-	-	-	-	1006759	19479	12684	3908	670489	14781	9392	3129	523087	9406	6066	2092
	Middle	-	-	-	-	-	-	-	-	2020083	10070	6175	1801	1757597	10479	6205	1812	1697485	9959	6167	1913
	High	-	-	-	-	-	-	-	-	597078	2685	1720	479	501802	2653	1588	521	538928	2425	1570	496

Di Girolamo C, *et al. Heart* 2019; 106:40–49. doi: 10.1136/heartjnl-2019-315129

Supplementary Table S 4. Distribution of person-years (py) and cardiovascular disease (CVD), ischaemic heart disease (IHD) and cerebrovascular disease (CeVD) deaths by educational level, period and population, women, 35-79 years

		1990-1994				1995-1999				2000-2004				2005-2009				2010-2014			
		**py**	**CVD**	**IHD**	**CeVD**	**py**	**CVD**	**IHD**	**CeVD**	**py**	**CVD**	**IHD**	**CeVD**	**py**	**CVD**	**IHD**	**CeVD**	**py**	**CVD**	**IHD**	**CeVD**
Finland	Low	3616611	21177	12019	5845	3166780	15959	9040	4371	2753025	12238	6653	3380	2285947	8626	4477	2223	1522433	5293	2443	1442
	Middle	1819277	2666	1387	835	1951567	2582	1340	755	2405151	2619	1221	830	2439843	2647	1202	771	2158583	2382	1046	671
	High	1229839	1108	511	381	1346273	1095	499	363	2037788	1044	454	361	2164519	1183	468	394	2248972	1129	402	370
Denmark	Low	-	-	-	-	2952647	9764	4632	2427	2808146	10494	4109	2981	2430715	7270	2538	2203	2109303	5213	1644	1548
	Middle	-	-	-	-	2058831	2380	998	692	2487065	3190	1083	1052	2780228	2720	819	925	2963329	2416	718	779
	High	-	-	-	-	1275369	788	287	263	1594865	1090	329	403	1947243	1015	282	370	2340507	1028	249	346
England and Wales	Low	547626	2468	1380	658	523884	2077	1112	538	551468	1543	753	425	522606	1055	480	282	286686	443	189	113
	Middle	-	-	-	-	-	-	-	-	-	-	-	-	-	-	-	-	-	-	-	-
	High	82117	113	47	39	87852	113	50	35	129357	117	44	45	133352	108	43	34	118696	64	23	15
Austria	Low	1089820	6672	2630	1798	-	-	-	-	945397	4510	1939	1083	-	-	-	-	1489246	3951	1742	792
	Middle	812279	2015	822	480	-	-	-	-	1103065	1734	761	371	-	-	-	-	2656966	2524	1083	519
	High	75478	95	38	20	-	-	-	-	161961	109	39	23	-	-	-	-	626690	242	82	49
Switzerland	Low	3085532	11613	4816	2402	2718748	9602	4108	2041	2473331	6043	2373	1294	2137069	4500	1637	994	1643671	2944	988	617
	Middle	3788293	4799	1935	1068	3893896	4746	1931	1044	4229886	3407	1254	812	4270400	3145	1107	776	3832595	2485	788	629
	High	505370	374	143	75	539677	379	147	95	889556	382	155	76	934549	414	132	97	884081	367	99	112
Spain, Barcelona	Low	1779565	4066	1211	1278	1584656	3384	1058	998	1439528	2655	723	739	1030970	1549	355	432	699172	859	213	228
	Middle	264286	216	57	70	323188	230	65	74	446065	254	66	77	407831	204	37	63	332507	155	27	46
	High	272812	145	39	57	342951	156	40	53	449393	183	46	62	442556	109	20	31	396912	104	25	28
Italy, Turin	Low	1050277	3088	876	1022	898806	2579	752	805	848741	2033	590	617	692559	1474	410	470	308043	579	168	160
	Middle	215215	229	73	80	213982	238	58	72	312374	251	58	81	298719	194	62	60	193946	111	28	29
	High	84653	67	20	24	84047	76	19	28	149738	118	33	37	146760	77	19	24	99599	23	4	9
Italy, Emilia	Low	-	-	-	-	-	-	-	-	581285	1411	493	367	459536	1005	327	281	439321	639	186	179
	Middle	-	-	-	-	-	-	-	-	278674	202	70	47	269081	153	53	42	394079	176	59	38
	High	-	-	-	-	-	-	-	-	157927	80	26	24	156742	67	18	27	256307	65	16	16
Hungary	Low	8366860	76388	27118	24077	-	-	-	-	7303436	66280	27954	19740	-	-	-	-	4506591	29068	14333	6656
	Middle	1900364	4339	1789	1237	-	-	-	-	2937424	5522	2401	1635	-	-	-	-	2802474	5174	2524	1126
	High	834204	1342	563	375	-	-	-	-	1244456	1632	700	473	-	-	-	-	1575303	1804	870	379
Poland	Low	-	-	-	-	-	-	-	-	15703887	105363	30832	31472	-	-	-	-	13495695	67796	17424	17374
	Middle	-	-	-	-	-	-	-	-	10485912	17538	5455	5317	-	-	-	-	11313549	19977	5115	5174
	High	-	-	-	-	-	-	-	-	3088419	2755	864	871	-	-	-	-	5109495	3750	927	1001
Estonia	Low	-	-	-	-	-	-	-	-	583644	8703	4576	2756	420061	5700	2617	1186	220858	2258	787	341
	Middle	-	-	-	-	-	-	-	-	1256349	4014	1930	1300	1266629	3852	1537	886	872204	2404	808	376
	High	-	-	-	-	-	-	-	-	415318	834	385	297	424250	902	365	233	428881	586	184	99
Lithuania	Low	-	-	-	-	-	-	-	-	1308298	17533	10031	5039	843691	13062	7405	3916	609265	8281	4948	2465
	Middle	-	-	-	-	-	-	-	-	2473355	5782	2942	1684	2170089	5954	2969	1750	2054878	5493	2940	1611
	High	-	-	-	-	-	-	-	-	822697	1223	630	385	714156	1242	600	417	834099	1299	644	413

Supplementary Table S 5. Distribution of person-years (py) and cardiovascular disease (CVD), ischeamic heart disease (IHD) and cerebrovascular disease (CeVD) deaths by occupational class, period and population, men, 35-64 years

		1990-1994				1995-1999				2000-2004				2005-2009				2010-2014			
		py	CVD	IHD	CeVD	py	CVD	IHD	CeVD	py	CVD	IHD	CeVD	py	CVD	IHD	CeVD	py	CVD	IHD	CeVD
Finland	Unknown or non-active	67118	276	173	42	73234	225	126	31	163572	349	203	51	178173	427	235	51	182988	382	183	48
	Upper non-manual employees	818206	1065	681	183	819347	927	593	140	963414	951	560	169	915425	900	500	125	881923	613	324	86
	Lower non-manual employees	871411	1736	1183	260	857153	1533	995	257	1037367	1581	968	264	984132	1441	850	202	854376	972	540	134
	Manual workers	2337156	7860	5291	1176	2203328	6551	4286	969	2469452	6190	3809	922	2282737	5894	3389	844	1791172	3994	2267	521
	Farmers	434793	1516	1058	203	359686	1068	701	182	311789	683	415	104	260977	599	355	97	159643	320	186	49
	Self-employed	477030	1048	677	172	450455	928	615	157	540128	922	553	152	487100	873	503	126	470016	600	305	96
Denmark	Unknown or non-active	-	-	-	-	1171109	4739	2651	848	1134691	4224	1988	880	1843198	4003	1672	867	1245851	2882	1186	590
	Upper non-manual employees	-	-	-	-	1388194	924	533	145	1446156	783	342	161	1531015	673	305	120	1698366	509	215	88
	Lower non-manual employees	-	-	-	-	402844	322	173	50	349068	275	136	45	366099	195	94	42	541307	262	126	43
	Manual workers	-	-	-	-	1657351	1506	920	249	2093402	1642	794	316	1458141	978	465	192	1777331	864	433	157
	Farmers	-	-	-	-	59444	46	33	4	35734	19	12	5	22426	14	9	1	24185	13	4	5
	Self-employed	-	-	-	-	504859	504	307	82	503164	400	196	75	505950	265	120	51	387800	145	68	22
England and Wales	Unknown or non-active	15011	67	48	10	14779	60	47	4	17999	54	34	11	17650	34	21	8	15166	38	23	6
	Upper non-manual employees	32974	54	39	5	32857	40	27	6	206068	190	129	25	198874	162	114	16	118742	68	50	7
	Lower non-manual employees	188588	327	228	44	183911	279	209	29	26556	28	19	1	25964	31	25	4	19769	18	10	3
	Manual workers	250154	717	554	83	237523	567	420	72	197047	374	265	43	181142	293	199	32	106762	118	70	16
	Self-employed									80784	115	71	19	71784	95	56	17	52880	35	26	3
Austria	Unknown or non-active	266856	2032	1144	317	-	-	-	-	319628	1495	844	210	-	-	-	-	658446	1695	981	212
	Non-manual employees	513745	484	327	53	-	-	-	-	640981	439	263	57	-	-	-	-	1360954	584	363	68
	Manual workers	441718	516	302	84	-	-	-	-	497158	428	272	56	-	-	-	-	1052391	623	389	81
	Farmers	59822	61	37	8	-	-	-	-	43542	29	16	3	-	-	-	-	-	-	-	-
	Self-employed	111642	161	88	32	-	-	-	-	144635	111	74	13	-	-	-	-	442759	194	126	25
Switzerland	Unknown or non-active	303089	1640	833	187	225461	722	364	102	1249757	2510	1280	283	1020064	1753	897	184	951142	1115	498	124
	Upper non-manual employees	2250925	2117	1299	174	2189646	2092	1249	187	2146281	1130	644	88	2089066	1169	670	99	1687463	819	436	60
	Lower non-manual employees	537592	769	439	72	532902	714	409	78	554922	393	224	37	551209	405	219	35	531156	334	167	37
	Manual workers	942746	1744	971	154	885491	1492	835	165	754420	729	384	78	726046	759	410	77	709737	606	312	47
	Farmers	279351	361	185	30	249301	346	171	29	214443	137	75	15	198844	155	84	15	157153	140	75	9
	Self-employed	598998	790	478	57	556906	760	429	77	629855	529	273	61	573368	519	315	46	420364	348	184	31
Italy, Turin	Unknown or non-active	40346	212	100	38	25143	91	33	19	63371	118	46	25	50913	98	52	14	-	-	-	-
	Upper non-manual employees	151271	152	76	32	127060	113	61	19	179806	112	49	17	148649	99	53	18	-	-	-	-
	Lower non-manual employees	175698	258	118	50	153005	146	79	11	155892	136	78	17	129946	93	50	19	-	-	-	-
	Manual workers	394565	705	330	145	308654	529	262	83	296461	332	162	52	236168	210	107	32	-	-	-	-
	Farmers	-	-	-	-	-	-	-	-	2063	5	2	1	1539	5	2	0	-	-	-	-
	Self-employed	149486	231	125	39	119039	184	93	28	135225	131	61	18	104603	97	49	12	-	-	-	-
Estonia	Unknown or non-active	-	-	-	-	-	-	-	-	414812	4430	2327	910	313183	2651	1200	369	299087	1967	812	233
	Upper non-manual employees	-	-	-	-	-	-	-	-	326235	514	304	95	321468	574	280	65	270562	205	96	25
	Lower non-manual employees	-	-	-	-	-	-	-	-	60421	105	56	22	62718	168	78	25	55214	72	32	2
	Manual workers	-	-	-	-	-	-	-	-	443517	1112	612	192	414476	1402	648	181	294485	454	221	31
	Farmers	-	-	-	-	-	-	-	-	118742	435	250	80	102732	418	210	56	52955	102	42	11
Lithuania	Unknown or non-active	-	-	-	-	-	-	-	-	1166076	9420	5567	1495	828388	7383	4185	1089	812390	7186	4210	1201
	Upper non-manual employees	-	-	-	-	-	-	-	-	399957	569	356	78	339200	658	367	108	406916	496	307	70
	Lower non-manual employees	-	-	-	-	-	-	-	-	106521	160	94	27	101953	178	103	35	120415	180	112	26
	Manual workers	-	-	-	-	-	-	-	-	869923	1979	1230	317	722667	2361	1351	387	661281	1232	726	199
	Farmers	-	-	-	-	-	-	-	-	92640	351	223	52	73837	352	202	51	28288	63	41	8
	Self-employed	-	-	-	-	-	-	-	-	317143	870	522	116	266082	967	543	145	177679	248	151	40

Di Girolamo C, *et al. Heart* 2019; 106:40–49. doi: 10.1136/heartjnl-2019-315129

Supplementary Table S 6.Population-Attributable Risk (PAR, per 100,000 person-year), Population-Attributable Fraction (PAF, %), Relative Index of Inequality (RII), Slope Index of Inequality (SII) with 95% confidence intervals (CI) of education for cardiovascular disease mortality between beginning of observation periods and 2010-14.

	PAR (100000pyrs)		PAF (%)		SII (95% CI)		RII (95% CI)	
	1990-94*	2010-2014	1990-94*	2010-2014	1990-94*	2010-2014	1990-94*	2010-2014
**Men**								
**Finland**	281.4 (270.5-292.0)	156.3 (153.5-159.3)	30.8 (29.4-32.3)	39.7 (38.5-40.9)	543.3 (506.1-580.4)	373.2 (355.2-391.2)	2.10 (2.00-2.20)	2.77 (2.63-2.92)
**Denmark**	198.3 (188.4-208.1)	88.0 (85.4-90.7)	32.9 (30.9-34.8)	35.4 (33.9-37.0)	297.9 (268.7-327.0)	215.2 (201.3-229.1)	1.86 (1.77-1.95)	2.60 (2.44-2.76)
**England and Wales**	241.3 (210.2-272.5)	64.6 (53.9-75.6)	30.2 (25.5-34.7)	26.1 (20.3-31.6)	546.5 (412.1-681.0)	199.8 (126.6-273.1)	2.40 (2.00-3.00)	2.40 (1.70-3.40)
**Austria**	269.8 (241.9-299.7)	107.6 (103.1-112.4)	33.8 (29.7-38.0)	35.2 (33.2-37.3)	297.9 (237.3-358.4)	225.6 (204.9-246.3)	1.45 (1.34-1.56)	2.08 (1.94-2.24)
**Switzerland**	140.9 (135.5-146.8)	67.7 (65.9-69.5)	26.3 (25.0-27.6)	32.6 (31.3-34.0)	322.7 (300.5-344.9)	186.6 (173.2-200.0)	1.89 (1.80-1.97)	2.79 (2.59-2.99)
**Spain (Barcelona)**	96.4 (84.3-109.8)	60.0 (53.6-66.4)	22.7 (19.3-26.2)	29.7 (25.3-33.7)	104.9 (63.7-146.2)	107.8 (77.5-138.2)	1.35 (1.21-1.50)	1.82 (1.53-2.16)
**Italy (Turin)**	104.5 (79.1-129.9)	39.6 (22.5-57.1)	19.2 (13.9-24.2)	18.8 (9.4-27.4)	145.1 (76.0-214.2)	68.6 (20.7-116.4)	1.37 (1.20-1.57)	1.38 (1.08-1.76)
**Italy (Emilia)**	61.5 (46.8-77.7)	41.7 (34.5-49.7)	20.3 (14.7-26.0)	28.3 (22.1-34.7)	120.3 (74.1-166.5)	99.9 (72.2-127.6)	1.60 (1.30-1.90)	2.10 (1.70-2.60)
**Hungary**	524.6 (512.4-537.4)	407.0 (402.5-411.2)	37.1 (36.0-38.2)	50.0 (49.1-51.0)	839.5 (798.2-880.9)	1005.0 (980.1-1029.9)	2.13 (2.06-2.20)	4.64 (4.45-4.84)
**Poland**	466.1 (461.6-470.6)	372.6 (369.9-375.5)	50.9 (50.2-51.7)	52.0 (51.5-52.7)	1013.3 (997.5-1029.2)	831.2 (822.1-840.2)	3.84 (3.76-3.93)	3.98 (3.92-4.05)
**Estonia**	508.6 (492.2-526.2)	338.3 (329.3-347.7)	40.6 (38.7-42.5)	43.7 (41.6-45.7)	1062.0 (1000.2-1123.9)	796.9 (741.6-852.3)	2.60 (2.46-2.76)	3.01 (2.77-3.27)
**Lithuania**	496.8 (483.8-508.9)	462.9 (455.2-470.6)	42.4 (40.9-43.8)	45.7 (44.5-46.9)	840.5 (798.5-882.6)	876.1 (835.2-917.0)	2.27 (2.17-2.37)	2.69 (2.56-2.83)
**Women**								
**Finland**	157.4 (149.5-166.1)	57.0 (54.9-59.2)	39.4 (37.0-41.9)	39.4 (37.3-41.6)	291.0 (269.2-312.8)	143.3 (132.5-154.2)	2.42 (2.25-2.60)	3.20 (2.92-3.51)
**Denmark**	127.4 (119.4-136.2)	48.0 (46.0-50.2)	42.9 (39.7-46.3)	38.4 (36.1-40.8)	225.6 (205.4-245.8)	127.7 (118.5-137.0)	2.88 (2.65-3.14)	3.59 (3.28-3.93)
**England and Wales**	130.1 (101.4-162.5)	46.0 (37.2-56.0)	32.0 (23.7-40.6)	34.7 (25.2-43.8)	276.2 (167.6-384.8)	123.2 (71.2-175.2)	2.70 (1.80-3.90)	3.10 (1.80-5.30)
**Austria**	154.5 (117.5-198.1)	56.1 (48.1-63.9)	36.0 (26.6-46.5)	37.4 (31.2-43.2)	249.7 (212.1-287.2)	105.2 (90.9-119.4)	1.87 (1.69-2.07)	2.14 (1.93-2.37)
**Switzerland**	84.7 (75.3-94.9)	32.3 (29.0-36.0)	37.1 (32.5-42.0)	35.2 (30.7-39.9)	170.4 (157.1-183.7)	73.7 (65.3-82.2)	2.27 (2.12-2.42)	2.57 (2.31-2.85)
**Spain (Barcelona)**	87.7 (78.2-97.9)	20.2 (14.4-26.9)	46.5 (40.2-52.8)	25.7 (16.8-34.8)	131.1 (103.8-158.4)	35.9 (16.4-55.5)	2.36 (1.89-2.94)	1.75 (1.31-2.35)
**Italy (Turin)**	83.6 (56.5-114.2)	42.6 (28.2-59.5)	32.7 (21.1-45.1)	42.7 (25.3-61.2)	140.9 (90.4-191.5)	73.7 (41.3-106.1)	2.00 (1.57-2.56)	2.32 (1.54-3.50)
**Italy (Emilia)**	29.0 (15.2-42.6)	17.5 (10.0-25.0)	22.1 (10.5-32.7)	25.2 (12.5-36.6)	49.6 (19.1-80.2)	34.4 (15.2-53.6)	1.50 (1.20-2.00)	1.70 (1.30-2.40)
**Hungary**	373.1 (357.0-388.7)	179.2 (173.7-186.0)	46.3 (44.0-48.5)	44.5 (42.8-46.3)	470.1 (433.4-506.8)	448.1 (432.4-463.8)	2.21 (2.09-2.34)	4.54 (4.30-4.80)
**Poland**	238.1 (232.6-243.5)	173.0 (170.5-175.4)	52.2 (50.7-53.6)	53.9 (52.8-54.9)	479.7 (469.5-489.9)	382.0 (376.7-387.2)	3.75 (3.63-3.87)	4.26 (4.17-4.36)
**Estonia**	249.4 (238.4-261.4)	129.8 (125.0-135.2)	44.2 (41.6-46.9)	42.6 (40.0-45.3)	546.2 (514.0-578.3)	342.1 (314.3-370.0)	3.17 (2.94-3.41)	3.62 (3.23-4.04)
**Lithuania**	255.2 (246.6-264.1)	199.6 (195.1-204.2)	47.2 (45.2-49.3)	48.4 (46.7-50.2)	434.5 (410.7-458.2)	413.8 (392.9-434.8)	2.66 (2.51-2.82)	3.46 (3.23-3.70)

* 1995-99 for Denmark, 2000-04 for Italy (Emilia), Poland, Estonia and Lithuania

Di Girolamo C, *et al. Heart* 2019; 106:40–49. doi: 10.1136/heartjnl-2019-315129

Supplementary Table S 7. Age-standardised cardiovascular disease mortality rates per 100,000 person years, by occupational class, period and population, men, 35-64 years

		1990-1994				1995-1999				2000-2004				2005-2009				2010-2014			
		ASMR	95% CI			ASMR	95% CI			ASMR	95% CI			ASMR	95% CI			ASMR	95% CI		
Finland	Upper non-manual employees	179.0	167.8	-	190.1	126.0	117.6	-	134.3	106.1	99.3	-	112.9	87.5	81.8	-	93.3	69.7	64.2	-	75.2
	Lower non-manual employees	249.5	237.6	-	261.5	188.1	178.5	-	197.7	164.4	156.2	-	172.5	133.9	127.0	-	140.9	112.6	105.6	-	119.7
	Manual workers	388.0	379.4	-	396.7	312.3	304.6	-	319.9	263.1	256.5	-	269.7	230.0	224.1	-	235.9	192.8	186.8	-	198.8
	Farmers	297.3	282.1	-	312.5	252.3	236.8	-	267.8	190.3	175.9	-	204.7	177.5	162.0	-	193.0	143.9	127.4	-	160.4
	Self-employed	262.9	246.6	-	279.1	205.4	191.8	-	218.9	169.6	158.5	-	180.8	144.2	134.0	-	154.3	109.7	100.9	-	118.6
Denmark*	Upper non-manual employees	-	-		-	116.7	108.7	-	124.7	91.4	84.7	-	98.1	70.3	65.0	-	75.7	49.6	45.3	-	53.9
	Lower non-manual employees	-	-		-	163.5	144.5	-	182.6	168.9	147.8	-	190.0	105.9	90.8	-	120.9	84.9	74.6	-	95.2
	Manual workers	-	-		-	210.4	199.0	-	221.8	160.5	152.5	-	168.6	147.8	138.4	-	157.1	108.7	101.4	-	116.0
	Farmers	-	-		-	168.1	117.8	-	218.3	162.5	87.9	-	237.2	221.0	100.5	-	341.4	163.9	74.4	-	253.5
	Self-employed	-	-		-	105.5	96.2	-	114.8	80.9	73.0	-	88.9	51.7	45.5	-	58.0	38.1	31.9	-	44.4
England and Wales	Upper non-manual employees	199.3	146.0	-	252.7	136.4	93.8		178.9	102.4	87.6	-	117.1	77.5	65.4		89.5	59.6	45.4	-	73.8
	Lower non-manual employees	196.4	175.1	-	217.8	156.5	138.0		175.0	114.3	71.7	-	156.9	117.9	76.4		159.4	90.4	48.6	-	132.1
	Manual workers	290.9	269.7	-	312.2	231.1	212.1		250.1	191.0	171.7	-	210.4	153.2	135.6		170.8	103.6	84.8	-	122.4
	Self-employed	-	-		-	-	-		-	138.1	112.8	-	163.4	111.8	89.2		134.3	61.6	41.2	-	82.0
Austria	Non-manual employees	119.3	104.0	-	134.6	-	-		-	82.7	72.6	-	92.8	-	-		-	53.5	48.6	-	58.4
	Manual workers	151.2	128.5	-	173.9	-	-		-	112.5	95.3	-	129.6	-	-		-	73.0	66.4	-	79.5
	Farmers†	111.3	75.9	-	146.6	-	-		-	90.5	47.5	-	133.5	-	-		-	-	-		-
	Self-employed	163.2	134.5	-	191.9	-	-		-	91.7	72.3	-	111.1	-	-		-	50.6	42.9	-	58.4
Switzerland*	Upper non-manual employees	121.7	116.5	-	127.0	102.1	97.7	-	106.6	74.0	69.6	-	78.4	62.1	58.5	-	65.7	50.6	47.1	-	54.1
	Lower non-manual employees	205.0	190.5	-	219.6	164.9	152.7	-	177.0	117.1	105.3	-	128.9	97.6	88.0	-	107.1	87.2	77.8	-	96.6
	Manual workers	233.1	222.2	-	244.1	189.3	179.7	-	198.9	154.4	143.1	-	165.7	136.5	126.7	-	146.3	114.1	105.0	-	123.2
	Farmers	128.8	115.5	-	142.2	131.0	117.1	-	144.9	78.6	65.4	-	91.7	81.7	68.6	-	94.8	87.5	72.7	-	102.3
	Self-employed	146.4	136.1	-	156.7	127.7	118.5	-	136.8	88.7	81.1	-	96.3	78.7	71.9	-	85.6	64.4	57.5	-	71.3
Italy (Turin)	Upper non-manual employees	105.0	87.8	-	122.2	73.3	59.5	-	87.1	61.5	49.9	-	73.1	56.6	45.2	-	67.9	-	-		-
	Lower non-manual employees	159.2	139.4	-	179.0	91.6	76.5	-	106.7	85.8	71.1	-	100.5	63.4	50.1	-	76.6	-	-		-
	Manual workers	152.0	140.5	-	163.5	133.3	121.3	-	145.3	103.0	91.6	-	114.4	77.7	66.9	-	88.6	-	-		-
	Farmers††	-	-		-	-	-		-	242.9	29.5	-	456.4	302.1	38.4	-	565.9	-	-		-
	Self-employed	143.7	124.9	-	162.6	124.4	105.7	-	143.0	89.5	73.8	-	105.2	79.6	63.2	-	96.1	-	-		-
Estonia*	Upper non-manual employees	-	-		-	-	-		-	294.8	268.8	-	320.8	229.4	210.3	-	248.6	149.0	128.2	-	169.8
	Lower non-manual employees	-	-		-	-	-		-	450.9	360.8	-	541.1	469.5	396.4	-	542.6	287.4	220.0	-	354.7
	Manual workers	-	-		-	-	-		-	581.7	547.0	-	616.5	503.2	476.1	-	530.3	354.5	321.5	-	387.4
	Farmers	-	-		-	-	-		-	424.1	384.4	-	463.9	384.5	347.4	-	421.5	208.8	168.2	-	249.3
Lithuania*	Upper non-manual employees	-	-		-	-	-		-	278.5	255.1	-	302.0	314.9	290.4	-	339.3	271.4	247.0	-	295.8
	Lower non-manual employees	-	-		-	-	-		-	400.3	333.4	-	467.1	409.5	345.6	-	473.4	389.4	329.2	-	449.5
	Manual workers	-	-		-	-	-		-	543.9	519.3	-	568.5	634.1	608.2	-	659.9	547.5	515.9	-	579.0
	Farmers	-	-		-	-	-		-	517.5	462.9	-	572.1	570.2	510.4	-	629.9	325.7	242.1	-	409.4
	Self-employed	-	-		-	-	-		-	432.0	401.9	-	462.1	489.4	457.8	-	521.1	218.0	189.2	-	246.7

* ASMRs are corrected for the exclusion of economically inactive

Supplementary Table S 8. Age-standardised ischaemic heart disease mortality rates per 100,000 person years, by occupational class, period and population, men, 35-64 years

		1990-1994		1995-1999		2000-2004		2005-2009		2010-2014	
		ASMR	95% CI	ASMR	95% CI	ASMR	95% CI	ASMR	95% CI	ASMR	95% CI
Finland	Upper non-manual employees	117.1	108.0 - 126.2	81.6	74.8 - 88.3	63.1	57.9 - 68.4	48.0	43.8 - 52.3	36.8	32.8 - 40.8
	Lower non-manual employees	172.9	162.9 - 182.9	123.5	115.8 - 131.3	100.9	94.5 - 107.3	78.6	73.3 - 83.9	62.4	57.2 - 67.7
	Manual workers	264.7	257.5 - 271.8	206.4	200.2 - 212.7	163.0	157.7 - 168.2	130.9	126.4 - 135.3	107.2	102.8 - 111.7
	Farmers	204.2	191.8 - 216.7	163.3	151.0 - 175.7	113.9	102.9 - 124.9	101.6	90.1 - 113.0	80.7	68.5 - 92.8
	Self-employed	173.6	160.3 - 187.0	136.8	125.7 - 147.8	101.4	92.8 - 110.0	80.0	72.8 - 87.2	54.9	48.7 - 61.1
Denmark*	Upper non-manual employees	-	- -	66.5	60.5 - 72.4	39.5	35.2 - 43.9	30.0	26.6 - 33.4	19.7	17.1 - 22.3
	Lower non-manual employees	-	- -	84.7	71.2 - 98.1	84.8	69.7 - 99.9	47.2	37.5 - 56.9	38.3	31.6 - 45.0
	Manual workers	-	- -	123.7	115.1 - 132.2	76.7	71.2 - 82.2	64.5	58.6 - 70.4	49.8	45.1 - 54.5
	Farmers	-	- -	113.8	73.7 - 153.9	100.9	42.5 - 159.2	129.1	41.7 - 216.4	46.3	0.6 - 92.0
	Self-employed	-	- -	63.6	56.4 - 70.8	39.5	33.9 - 45.1	22.9	18.8 - 27.0	17.8	13.6 - 22.0
England and Wales	Upper non-manual employees	143.8	98.6 - 189.1	90.4	56.1 - 124.7	69.7	57.5 - 81.8	54.5	44.4 - 64.5	44.2	32.0 - 56.5
	Lower non-manual employees	137.9	119.9 - 155.9	117.6	101.5 - 133.6	81.0	44.5 - 117.6	94.6	57.5 - 131.7	50.6	19.2 - 81.9
	Manual workers	224.7	206.0 - 243.4	171.0	154.7 - 187.4	135.4	119.1 - 151.7	103.2	88.8 - 117.5	61.1	46.7 - 75.5
	Self-employed	-	- -	-	- -	85.1	65.2 - 104.9	66.6	49.1 - 84.1	45.9	28.2 - 63.5
Austria	Non-manual employees	79.9	67.4 - 92.4	-	- -	50.3	42.4 - 58.2	-	- -	33.8	29.8 - 37.7
	Manual workers	91.2	73.1 - 109.4	-	- -	69.4	56.4 - 82.4	-	- -	45.8	40.6 - 51.0
	Farmers†	66.5	39.2 - 93.8	-	- -	58.2	21.4 - 95.1	-	- -	-	- -
	Self-employed	84.3	64.5 - 104.1	-	- -	63.7	47.0 - 80.3	-	- -	32.7	26.5 - 38.9
Switzerland*	Upper non-manual employees	73.0	69.0 - 77.0	60.4	57.0 - 63.8	41.4	38.1 - 44.7	34.3	31.7 - 36.9	25.7	23.3 - 28.2
	Lower non-manual employees	112.2	101.6 - 122.7	92.5	83.4 - 101.5	63.6	55.1 - 72.1	49.8	43.2 - 56.5	41.2	34.9 - 47.5
	Manual workers	124.9	117.0 - 132.8	103.1	96.1 - 110.1	78.0	70.1 - 85.9	69.8	63.1 - 76.6	54.6	48.5 - 60.6
	Farmers	64.6	55.2 - 73.9	64.4	54.7 - 74.1	42.4	32.8 - 52.0	42.7	33.5 - 52.0	45.2	34.7 - 55.8
	Self-employed	88.5	80.5 - 96.5	71.8	65.0 - 78.6	45.6	40.1 - 51.0	46.9	41.7 - 52.1	34.1	29.1 - 39.1
Italy (Turin)	Upper non-manual employees	52.5	40.3 - 64.6	39.6	29.4 - 49.7	26.7	19.1 - 34.4	30.1	21.9 - 38.4	-	- -
	Lower non-manual employees	72.8	59.4 - 86.2	49.6	38.4 - 60.8	49.4	38.2 - 60.5	33.8	24.2 - 43.3	-	- -
	Manual workers	71.4	63.5 - 79.3	65.0	56.7 - 73.3	50.6	42.6 - 58.6	39.5	31.8 - 47.2	-	- -
	Farmers††					92.9	0.0 - 221.7	121.2	0.0 - 288.9	-	- -
	Self-employed	77.9	64.0 - 91.8	60.9	48.3 - 73.5	41.8	31.1 - 52.6	39.1	27.9 - 50.3	-	- -
Estonia*	Upper non-manual employees	-	- -	-	- -	168.4	149.1 - 187.7	109.9	96.7 - 123.1	67.1	53.4 - 80.7
	Lower non-manual employees	-	- -	-	- -	228.5	165.7 - 291.2	213.6	164.8 - 262.5	122.5	79.5 - 165.5
	Manual workers	-	- -	-	- -	301.2	276.9 - 325.4	225.1	207.3 - 242.8	155.0	134.3 - 175.6
	Farmers	-	- -	-	- -	239.5	209.8 - 269.1	190.9	165.1 - 216.7	82.7	57.7 - 107.8
Lithuania*	Upper non-manual employees	-	- -	-	- -	170.9	152.7 - 189.1	175.5	157.2 - 193.8	166.4	147.4 - 185.4
	Lower non-manual employees	-	- -	-	- -	234.0	183.3 - 284.8	243.0	193.4 - 292.6	234.5	188.8 - 280.3
	Manual workers	-	- -	-	- -	325.1	306.4 - 343.7	364.1	344.5 - 383.7	311.1	287.7 - 334.4
	Farmers	-	- -	-	- -	329.8	286.1 - 373.4	326.7	281.5 - 371.9	214.4	146.1 - 282.7
	Self-employed	-	- -	-	- -	263.3	239.7 - 287.0	280.7	256.5 - 304.9	132.3	109.9 - 154.7

* ASMRs are corrected for the exclusion of economically inactive

Di Girolamo C, *et al. Heart* 2019; 106:40–49. doi: 10.1136/heartjnl-2019-315129

Supplementary Table S 9. Age-standardised cerebrovascular disease mortality rates per 100,000 person years, by occupational class, period and population, men, 35-64 years

		1990-1994				1995-1999				2000-2004				2005-2009				2010-2014			
		ASMR	95% CI			ASMR	95% CI			ASMR	95% CI			ASMR	95% CI			ASMR	95% CI		
Finland	Upper non-manual employees	30.0	25.5	-	34.6	18.9	15.7	-	22.1	18.9	16.0		21.7	12.2	10.0	-	14.4	9.8	7.7	-	11.8
	Lower non-manual employees	36.7	32.1	-	41.2	30.7	26.9	-	34.5	27.5	24.2	-	30.9	18.8	16.2	-	21.5	15.4	12.8	-	18.0
	Manual workers	56.8	53.5	-	60.1	45.7	42.8	-	48.6	39.0	36.5	-	41.6	33.3	31.0	-	35.6	25.3	23.1	-	27.5
	Farmers	40.3	34.7	-	46.0	44.1	37.5	-	50.7	29.3	23.6	-	35.0	27.8	22.2	-	33.5	23.9	16.9	-	30.9
	Self-employed	41.0	34.7	-	47.3	34.7	29.1	-	40.3	28.3	23.7	-	32.8	22.5	18.0	-	27.0	17.3	13.8	-	20.9
Denmark*	Upper non-manual employees	-	-		-	18.0	14.9	-	21.1	19.6	16.4	-	22.8	13.3	10.9	-	15.7	9.3	7.3	-	11.2
	Lower non-manual employees	-	-		-	26.1	18.4	-	33.9	28.1	19.4	-	36.7	24.4	16.9	-	31.9	15.0	10.5	-	19.6
	Manual workers	-	-		-	36.4	31.5	-	41.2	32.0	28.3	-	35.6	31.8	27.2	-	36.3	22.0	18.5	-	25.5
	Farmers	-	-		-	14.0	0.1	-	27.9	44.3	4.6	-	84.1	11.0	0.0	-	32.4	72.5	8.6	-	136.5
	Self-employed	-	-		-	16.9	13.2	-	20.6	15.1	11.6	-	18.5	10.1	7.3	-	12.9	5.8	3.4	-	8.2
England and Wales	Upper non-manual employees	18.1	2.1	-	34.2	22.1	4.2	-	39.9	13.3	8.0	-	18.6	8.3	4.1	-	12.4	6.0	1.6	-	10.5
	Lower non-manual employees	26.2	18.4	-	34.0	16.3	10.3	-	22.3	3.9	0.0	-	11.7	15.4	0.3	-	30.5	14.5	0.0	-	31.0
	Manual workers	33.7	26.5	-	41.0	29.5	22.7	-	36.3	22.0	15.4	-	28.6	16.9	11.0	-	22.9	14.0	7.2	-	20.9
	Self-employed	-	-		-	-	-		-	22.6	12.4	-	32.8	19.5	10.2	-	28.7	5.3	0.0	-	11.3
Austria	Non-manual employees	14.2	8.6	-	19.8	-	-		-	11.2	7.3	-	15.1	-	-		-	6.5	4.7	-	8.2
	Manual workers	20.3	13.4	-	27.2	-	-		-	14.0	8.2	-	19.9	-	-		-	8.6	6.5	-	10.8
	Farmers†	12.9	1.3	-	24.4	-	-		-	6.6	0.0	-	14.4	-	-		-				
	Self-employed	36.1	21.8	-	50.3	-	-		-	11.7	4.4	-	18.9	-	-		-	6.5	3.7	-	9.3
Switzerland*	Upper non-manual employees	10.3	8.7	-	11.9	9.6	8.2	-	11.0	6.0	4.7	-	7.3	5.6	4.5	-	6.7	4.2	3.1	-	5.2
	Lower non-manual employees	20.4	15.7	-	25.2	19.6	15.2	-	24.0	12.6	8.5	-	16.8	8.9	5.9	-	11.8	10.9	7.4	-	14.5
	Manual workers	22.4	18.8	-	25.9	22.6	19.2	-	26.1	18.1	14.0	-	22.2	15.0	11.6	-	18.4	10.4	7.5	-	13.4
	Farmers	11.4	7.3	-	15.5	11.5	7.2	-	15.8	8.9	4.4	-	13.4	7.7	3.8	-	11.7	6.5	2.2	-	10.9
	Self-employed	10.4	7.7	-	13.1	13.1	10.1	-	16.0	10.5	7.8	-	13.1	7.1	5.0	-	9.1	5.4	3.5	-	7.4
Italy (Turin)	Upper non-manual employees	22.4	14.4	-	30.4	12.2	6.5	-	17.9	9.1	4.6	-	13.5	10.2	5.3	-	15.0	-	-		-
	Lower non-manual employees	30.7	22.0	-	39.4	7.2	3.0	-	11.5	10.5	5.4	-	15.7	12.6	6.8	-	18.5	-	-		-
	Manual workers	31.2	26.0	-	36.4	20.3	15.8	-	24.9	15.9	11.5	-	20.4	11.4	7.4	-	15.5	-	-		-
	Farmers††									44.1	0.0	-	130.4	0.0	0.0	-	0.0	-	-		-
	Self-employed	24.8	16.9	-	32.7	19.1	11.8	-	26.3	12.3	6.5	-	18.2	11.1	4.5	-	17.7	-	-		-
Estonia*	Upper non-manual employees	-	-		-	-	-		-	57.2	45.5	-	68.9	26.5	20.0	-	33.0	20.8	12.4	-	29.2
	Lower non-manual employees	-	-		-	-	-		-	100.2	56.3	-	144.0	75.5	44.9	-	106.2	10.2	0.0	-	24.7
	Manual workers	-	-		-	-	-		-	110.2	94.3	-	126.1	69.3	58.9	-	79.7	31.6	20.4	-	42.8
	Farmers	-	-		-	-	-		-	80.1	62.5	-	97.6	51.9	38.3	-	65.4	24.5	10.0	-	39.0
Lithuania*	Upper non-manual employees	-	-		-	-	-		-	38.1	29.4	-	46.8	48.4	39.1	-	57.7	38.2	29.1	-	47.4
	Lower non-manual employees	-	-		-	-	-		-	78.7	47.5	-	109.9	78.8	51.4	-	106.2	63.9	37.8	-	90.0
	Manual workers	-	-		-	-	-		-	87.1	77.2	-	96.9	97.8	87.9	-	107.7	91.8	78.6	-	105.0
	Farmers	-	-		-	-	-		-	76.3	55.3	-	97.3	81.4	58.9	-	103.9	38.3	10.4	-	66.1
	Self-employed	-	-		-	-	-		-	58.8	47.5	-	70.1	73.9	61.5	-	86.2	38.3	25.7	-	50.9

* ASMRs are corrected for the exclusion of economically inactive

Di Girolamo C, *et al. Heart* 2019; 106:40–49. doi: 10.1136/heartjnl-2019-315129

Supplementary Table S 10. Relative and absolute annualised changes in age-standardised mortality rates between end and beginning of observation periods for cardiovascular, ischaemic and cerebrovascular disease mortality, by upper non-manual and manual occupation, men, 35-64 years

		**Cardiovascular disease**		**Ischaemic heart disease**		**Cerebrovascular disease**	
		relative	absolute	relative	absolute	relative	absolute
Finland	Upper non-manual employees	-4.8	-5.7	-5.9	-4.2	-5.7	-1.1
	Manual workers	-3.6	-10.3	-4.6	-8.3	-4.2	-1.7
Denmark*	Upper non-manual employees	-5.5	-4.5	-7.8	-3.1	-4.3	-0.6
	Manual workers	-4.3	-6.8	-5.9	-4.9	-3.3	-1.0
England and Wales	Upper non-manual employees	-6.5	-7.8	-6.3	-5.5	-5.9	-0.7
	Manual workers	-5.6	-10.4	-7.0	-9.1	-4.8	-1.1
Austria	Non-manual employees	-3.9	-3.3	-4.2	-2.3	-3.9	-0.4
	Manual workers	-3.6	-3.9	-3.4	-2.3	-4.2	-0.6
Switzerland*	Upper non-manual employees	-4.5	-3.7	-5.3	-2.5	-4.7	-0.3
	Manual workers	-3.7	-6.3	-4.3	-3.7	-3.9	-0.6
Italy (Turin)**	Upper non-manual employees	-3.8	-6.6	-3.4	-1.4	-4.8	-1.4
	Manual workers	-4.1	-9.5	-3.6	-2.0	-6.1	-1.9
Estonia*	Upper non-manual employees	-6.0	-13.3	-8.0	-9.2	-8.8	-3.3
	Manual workers	-4.4	-20.7	-5.9	-13.3	-10.7	-7.1
Lithuania*	Upper non-manual employees	-0.3	-0.7	-0.3	-0.4	0.0	0.0
	Manual workers	0.1	0.4	-0.4	-1.4	0.5	0.5

* Age-standardised morytality rates are corrected for the exclusion of economically inactive,** Annualised changes between 1990-94 and 2005-09

Di Girolamo C, *et al. Heart* 2019; 106:40–49. doi: 10.1136/heartjnl-2019-315129

Supplementary Table S 11. Population-Attributable Risk (PAR, per 100,000 person-year) and Population-Attributable Fraction (PAF, %) of occupational class for cardiovascular disease mortality, ischaemic heart disease mortality and cerebrovascular disease mortality between beginning of observation periods and 2010-14

	PAR (100000pyrs)		PAF (%)		AID (absolute version)		AID (relative version)	
	1990-94*	2010-2014**	1990-94*	2010-2014**	1990-94*	2010-2014**	1990-94*	2010-2014**
					**Cardiovascular disease**			
**Finland**	136.8	75.8	43.3	52.1	43.8	27.1	14.2	19.5
**Denmark**	57.2	32.6	32.9	39.7	24.3	15.8	15.2	20.4
**England and Wales**	57.2	29.4	22.3	33.0	23.5	10.5	9.5	13.6
**Austria**	115.2	39.1	49.1	42.3	5.9	4.9	4.9	8.2
**Switzerland**	41.8	19.5	25.5	27.8	23.5	13.3	14.9	18.3
**Italy (Turin)**	50.8	24.5	32.6	30.3	8.6	5.5	6.0	7.7
**Estonia**	221.2	145.5	42.9	49.4	68.4	50.5	15.0	19.8
**Lithuania**	234.6	173.7	45.7	39.0	55.4	74.3	12.2	18.3
					**Ischaemic heart disease**			
**Finland**	97.9	42.6	45.5	53.6	30.5	15.7	14.5	20.7
**Denmark**	33.6	16.0	33.6	44.9	14.4	7.6	15.6	22.2
**England and Wales**	47.9	13.5	25.0	23.3	21.6	4.0	11.7	7.8
**Austria**	56.0	21.9	41.2	39.3	3.7	2.9	5.0	7.7
**Switzerland**	19.8	9.1	21.3	26.1	11.5	6.1	12.9	16.9
**Italy (Turin)**	22.3	11.3	29.9	27.2	3.8	2.2	5.4	6.1
**Estonia**	110.9	59.6	39.7	47.1	31.6	21.7	13.0	19.5
**Lithuania**	139.4	97.6	44.9	37.0	32.5	39.5	11.8	16.6
					**Cerebrovascular disease**			
**Finland**	16.6	10.0	35.6	50.7	5.8	3.4	12.8	17.8
**Denmark**	11.5	6.6	39.0	41.6	4.7	3.7	17.9	24.2
**England and Wales**	13.1	5.2	41.8	46.3	2.4	2.1	8.1	22.4
**Austria**	21.1	5.1	59.8	43.9	3.0	0.5	16.1	6.7
**Switzerland**	4.4	2.3	29.9	36.0	2.5	1.5	18.2	22.6
**Italy (Turin)**	8.0	2.6	26.4	20.6	1.8	0.4	6.3	4.0
**Estonia**	45.1	10.9	44.1	34.3	12.7	3.5	14.5	13.9
**Lithuania**	42.7	35.8	52.8	48.3	10.5	13.3	15.0	20.2

* 1995-99 for Denmark, 2000-04 for Estonia and Lithuania, **2005-09 for Italy (Turin)

Di Girolamo C, *et al. Heart* 2019; 106:40–49. doi: 10.1136/heartjnl-2019-315129

Supplementary Figure S 1.Trend in ischaemic heart disease mortality (ASMR: age-standardised mortality rates) and inequalities (Relative and Slope Index of Inequality) in mortality by educational level, population and gender, 35-79 years

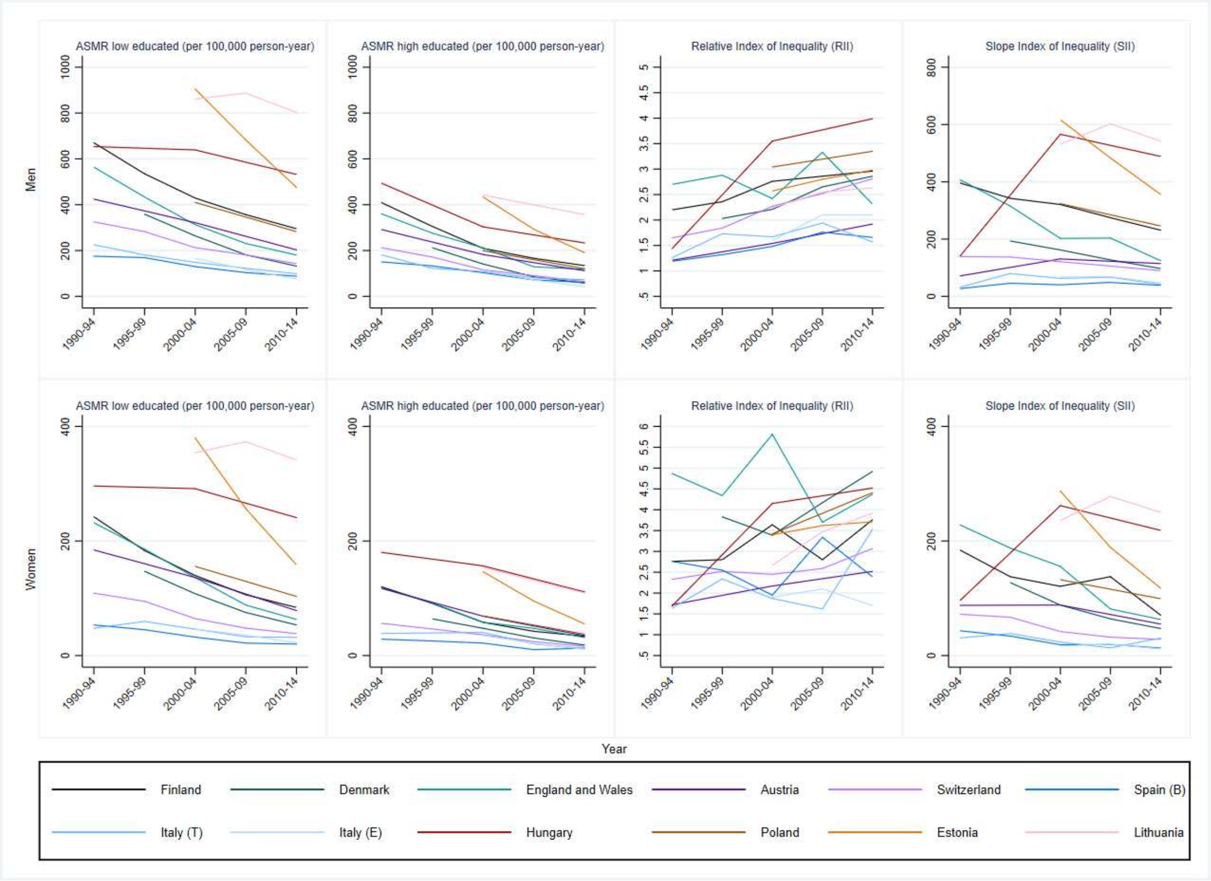

Di Girolamo C, *et al. Heart* 2019; 106:40–49. doi: 10.1136/heartjnl-2019-315129

Supplementary Figure S 2. Trend in cerebrovascular disease mortality (ASMR: age-standardised mortality rates) and inequalities (Relative and Slope Index of Inequality) in mortality by educational level, population and gender, 35-79 years

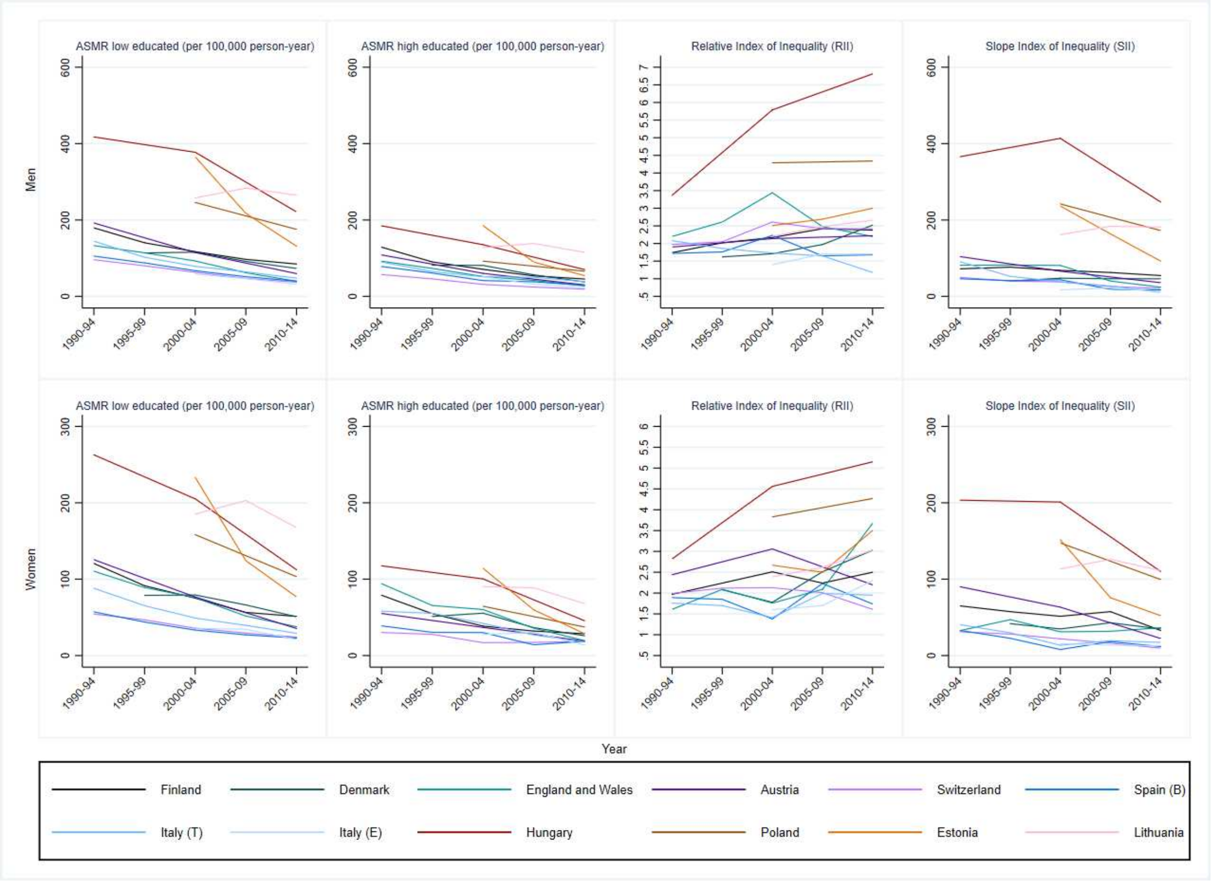

Supplementary Figure S 3. Changes in absolute and relative educational inequalities in ischaemic heart disease mortality between 2000-04 (period in which data became available for all populations) and 2010-14, by population and gender, 35-79 years

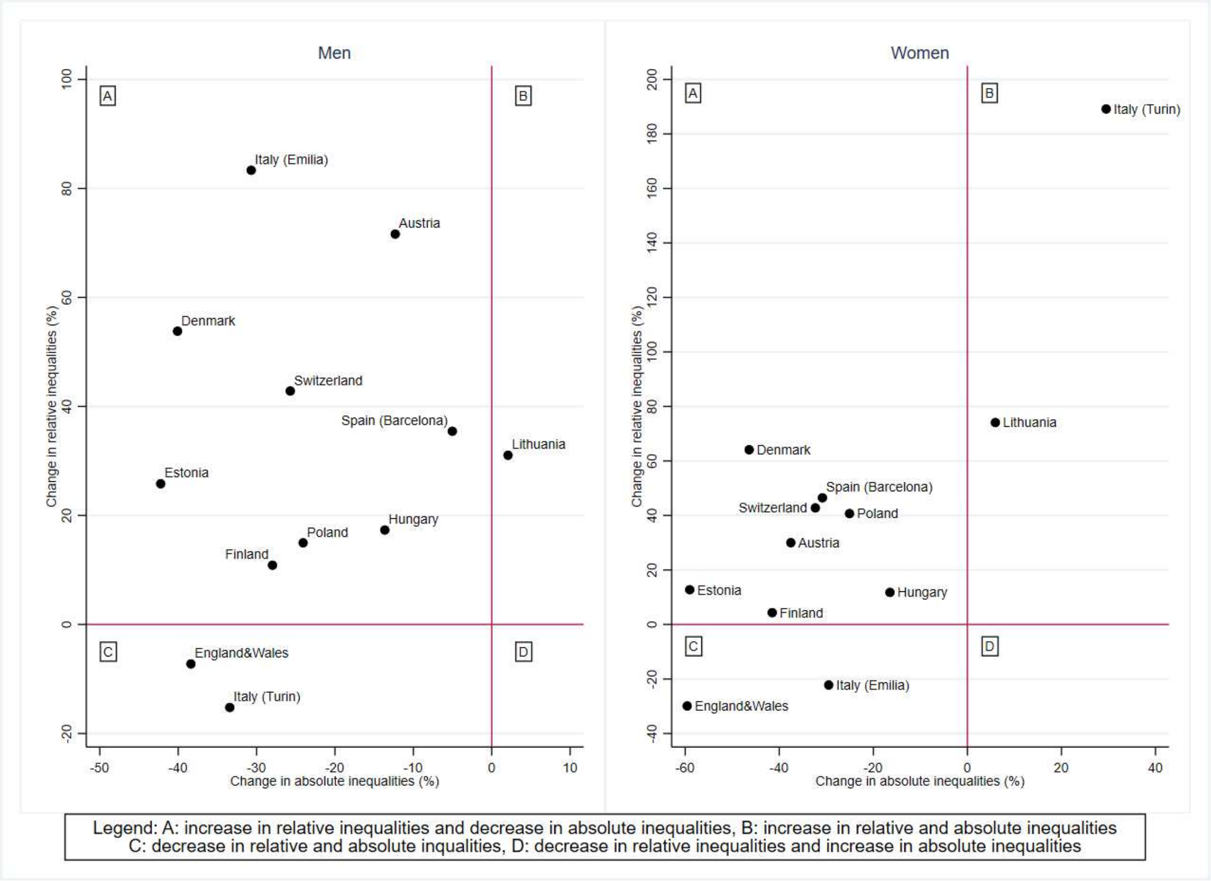

Note: changes in relative inequalities (ratio measures) were calculated using the following formula where RII stands for Relative Index of Inequality: $100*(RII_{2010\text{-}14}-RII_{2000\text{-}04})/(RII_{2000\text{-}04} - 1)$. Changes in absolute inequalities (difference measures) were calculated using the following formula where SII stands for Slope Index of Inequality: $100*(SII_{2010\text{-}14}-SII_{2000\text{-}04})/(SII_{2000\text{-}04})$.

Di Girolamo C, *et al. Heart* 2019; 106:40–49. doi: 10.1136/heartjnl-2019-315129

Supplementary Figure S 4. Changes in absolute and relative educational inequalities in cerebrovascular disease mortality between 2000-04 (period in which data became available for all populations) and 2010-14, by population and gender, 35-79 years

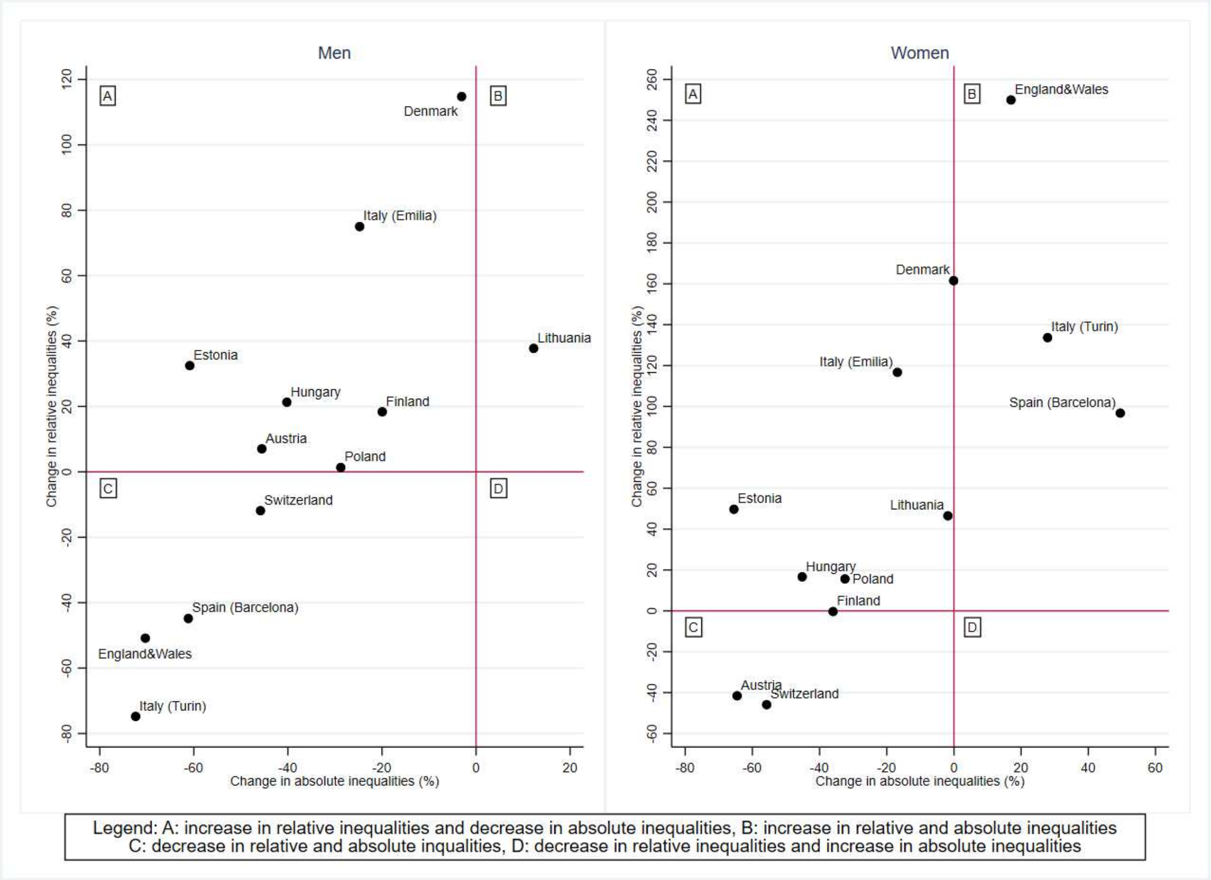

Note: changes in relative inequalities (ratio measures) were calculated using the following formula where RII stands for Relative Index of Inequality: $100*(RII_{2010-14}-RII_{2000-04})/(RII_{2000-04} - 1)$. Changes in absolute inequalities (difference measures) were calculated using the following formula where SII stands for Slope Index of Inequality: $100*(SII_{2010-14}-SII_{2000-04})/(SII_{2000-04})$.

Supplementary Figure S 5. Trends in cardiovascular disease mortality (ASMR: age-standardised mortality rates) and inequalities (Average Inter-group Difference, relative and absolute version) by occupation class, men, 35-64 years

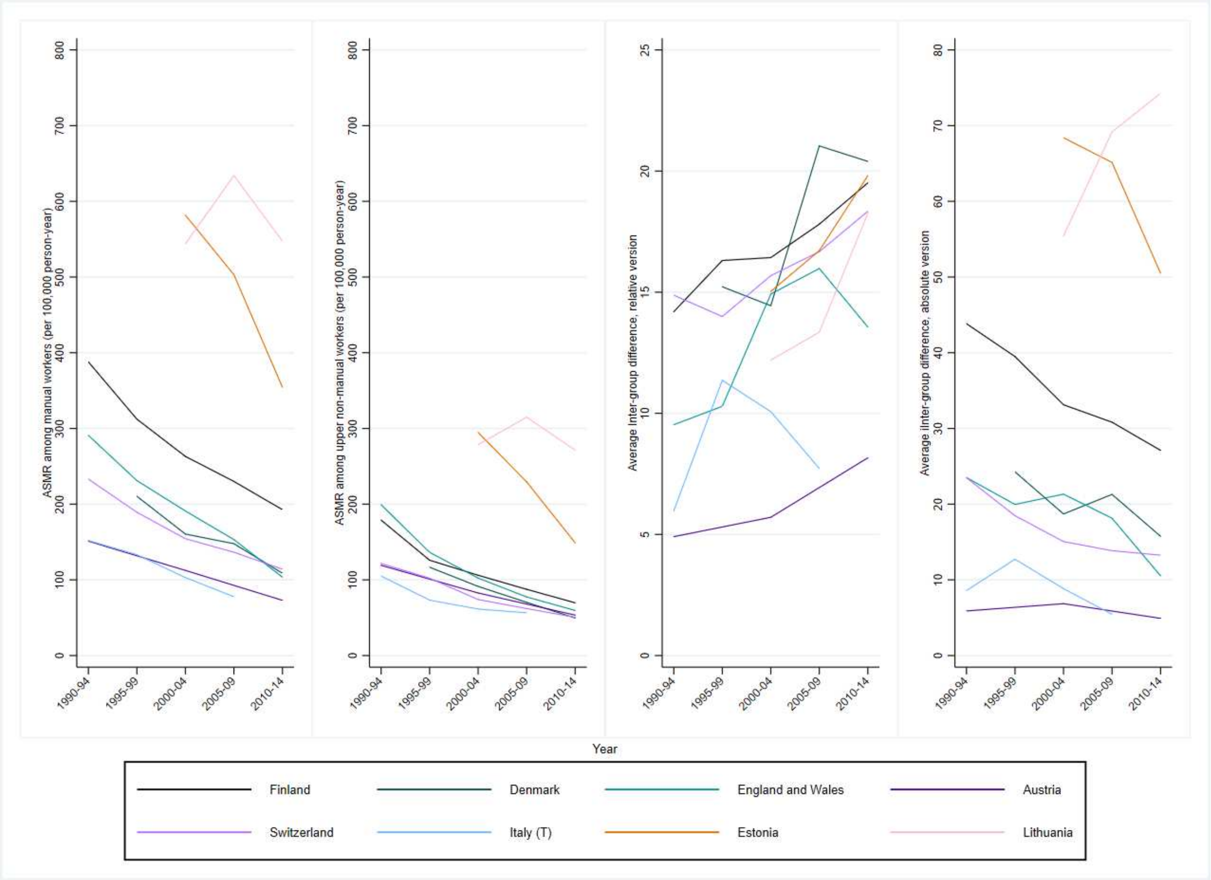

Note: AID relative and absolute versions were computed based on five occupational classes (upper non-manual, lower non-manual, manual, farmers and self-employed; for England and Wales self-employed was not available before 2000, for Austria upper non-manual and lower non-manual were grouped and farmers were only available for the years 1991 and 2001, for Estonia the category self-employed was not available).

Supplementary Figure S 6. Trends in inequalities (Relative and Slope Index of Inequality) for ischaemic heart disease and other heart diseases mortality by educational level, population and gender, 36-79 years

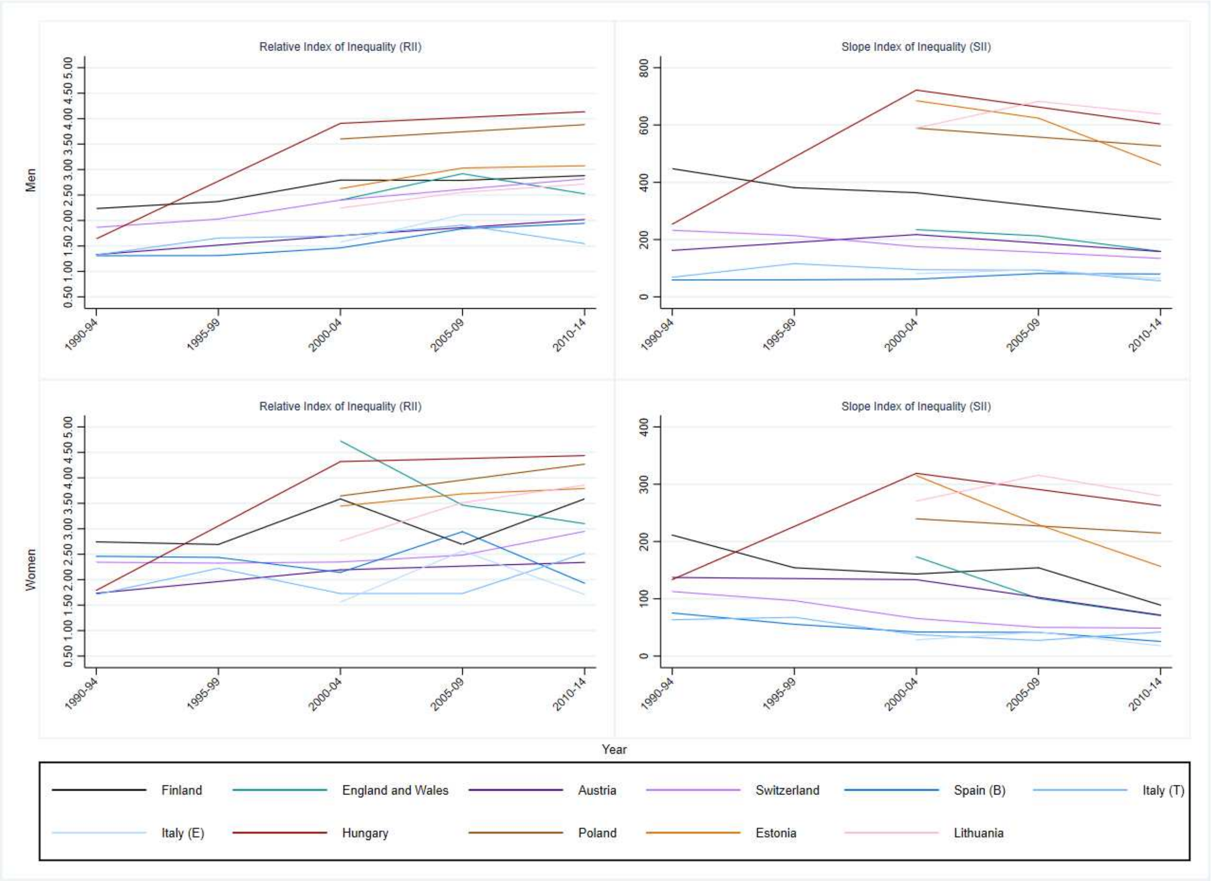

Note: For Denmark, it was not possible to estimate the RII and the SII for ischaemic and other heart diseases combined because the second group was not available in the data; for England and Wales information on other heart diseases were available since 2001.
